# Supplementary material for: Combine antibody-drug conjugate with cmLumiOpto for triple-negative breast cancer treatment
Source: Cell Rep Med. 2026 Jul 24;7(8):102934. doi: 10.1016/j.xcrm.2026.102934 (PMC13522777; doi:10.1016/j.xcrm.2026.102934)
Supplement: Supplementary file 1 [file mmc1.pdf]

Cell Reports Medicine, Volume *7*

Supplemental information

Combine antibody-drug conjugate with cmLumiOpto

for triple-negative breast cancer treatment

Tanvi Varadkar, Zhuoxin "Zora" Zhou, Yingnan Si, Jiashuai Zhang, Zhantao Du, Anjana Gautam Khanal, Srijita Chowdhury, Chandini Tsaliki, Lufang Zhou, and Xiaoguang "Margaret" Liu

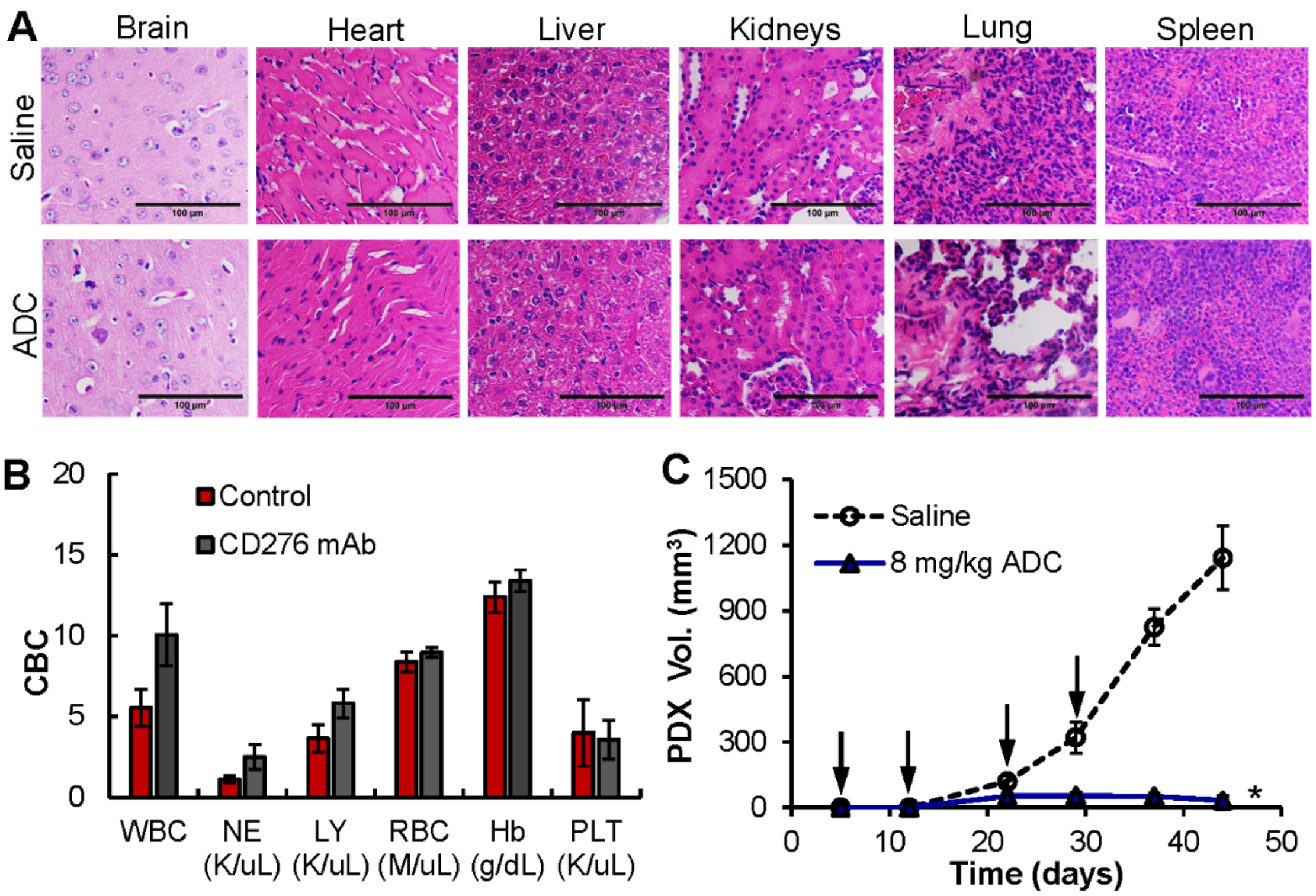

Figure S1. Evaluations CD276 mAb and ADC. Related to Figure 3. (**A**) H&E staining of major organs (brain, heart, liver, kidney, lung, and spleen) at 28 days post i.v. injection of ADC *via* tail vein in BALB/cJ mice. Scale bar: 100 µm. (**B**) Whole blood analysis showing minimal effect of CD276 mAb on blood cell count and peripheral immunity in BALB/cJ. n=4. Data are presented as mean±SEM. WBC: white blood cell; NE: neutrophils; LY: lymphocyte; RBC: red blood cell; Hb: hemoglobin; PTL: platelet. (**C**) PDX tumor volume profile. TNBC PDX xenografted female NSG mice treated with CD276 mAb-DM1 and saline following schedule of Q4Dx4 as indicated by the black arrow. n=7 mice/group. Data are presented as mean±SEM. *P<0.05 *vs*. saline using ANOVA followed by Dunnett's *t*-test.

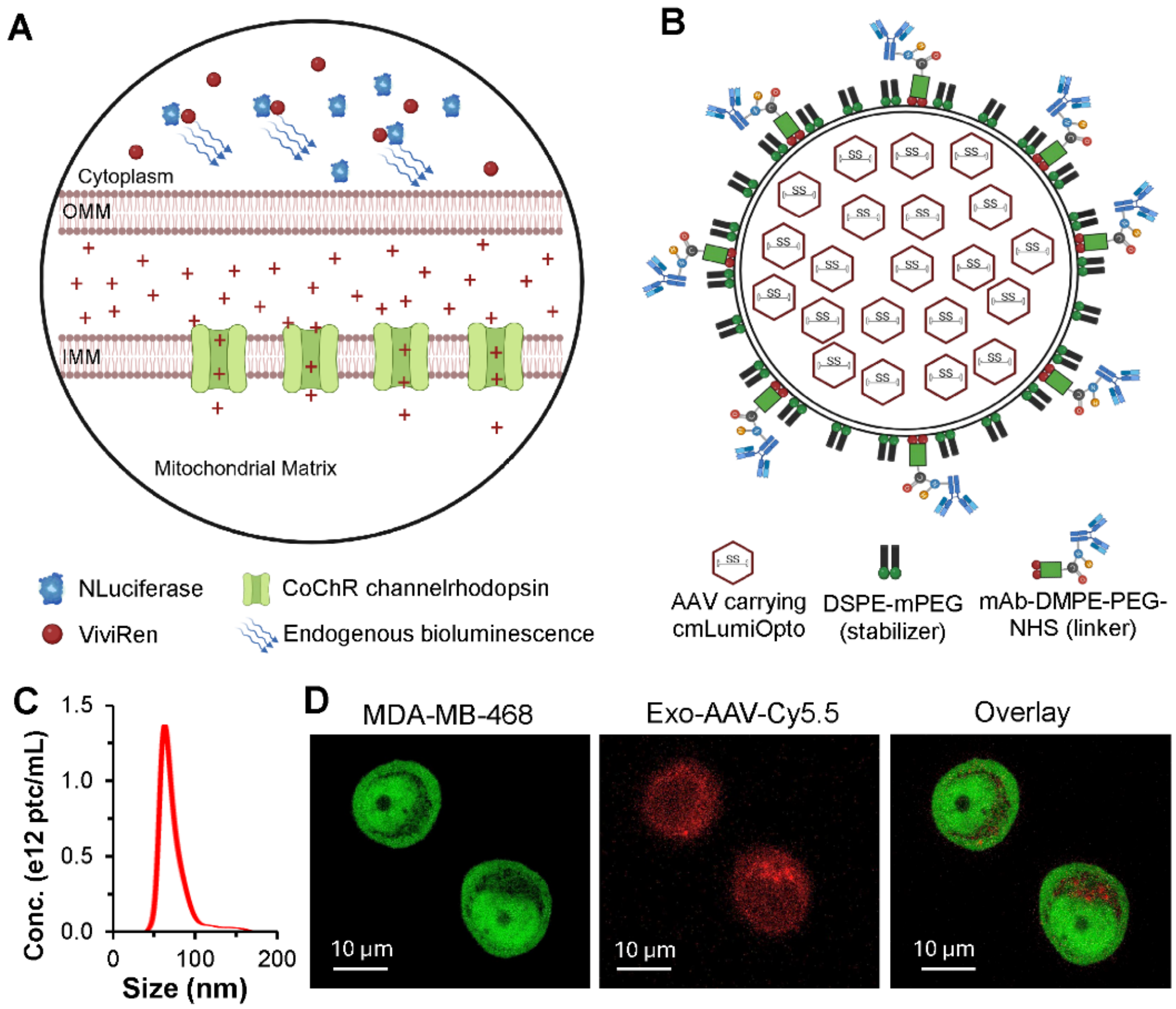

Figure S2. Construction and characterization of mAb-Exo-AAV. Related to Figure 4. (**A**) Diagram of the concept of cmLumiOpto gene therapy. cmLumiOpto gene vector leads to the expression of the CoChR protein with *cfos* promoter in the inner mitochondrial membrane of cancer mitochondria. ViviRen coupled with NLuc in cell cytoplasm generates a bright endogenous luminescent signal that activates the CoChR channels and causes depolarization of cancer mitochondria. (**B**) Structure of CD276 mAb-Exo-AAV which delivers cmLumiOpto gene. (**C**) Nanosight Pro analysis the concentration and particle size distribution of mAb-Exo-AAV. (**D**) Confocal image showing the internalization of mAb-Exo-AAV into MDA-MB-468 cells 24 hrs after incubation.

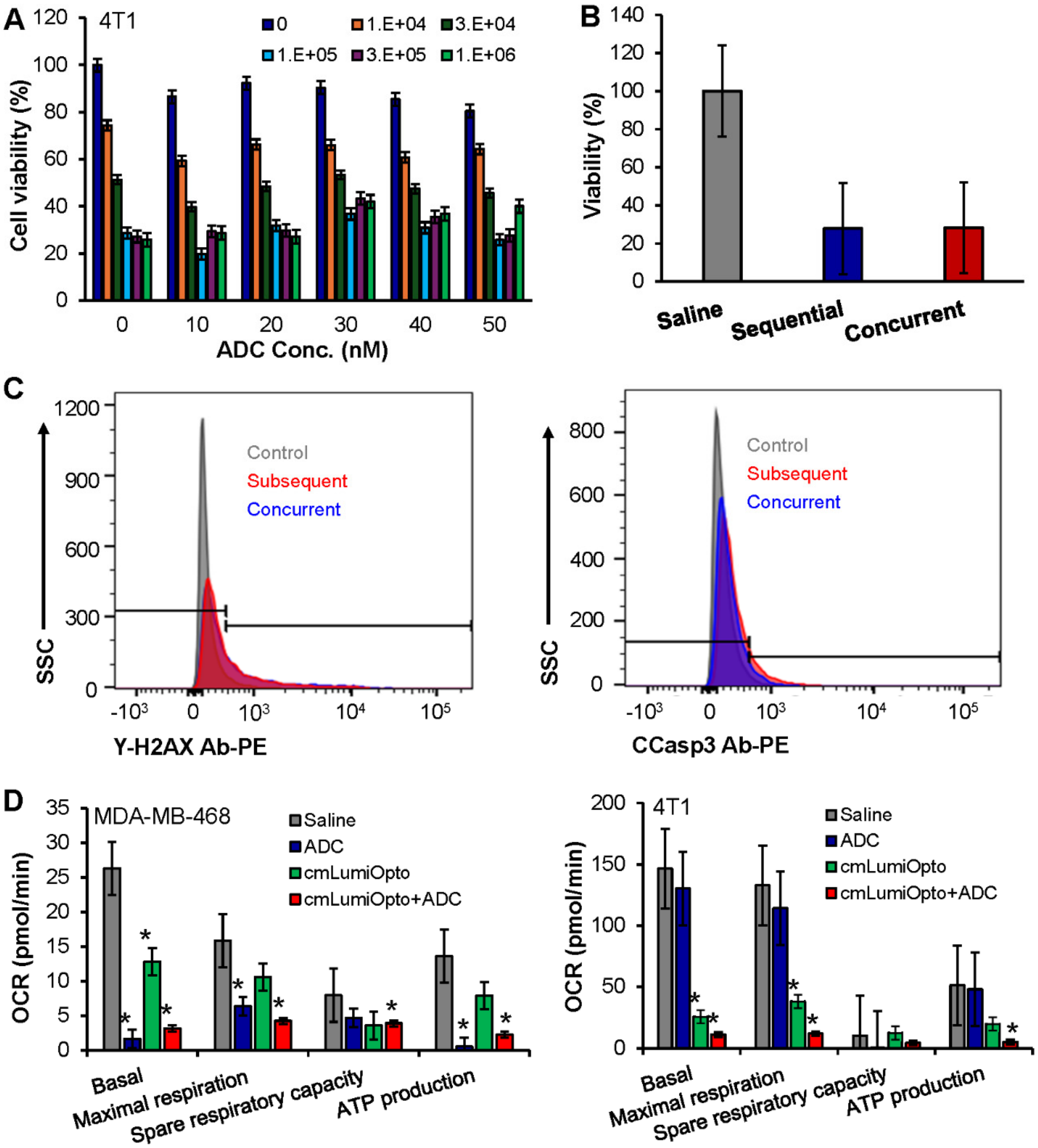

Figure S3. ***In vitro*** **assessment of synergism of cmLumiOpto and ADC. Related to Figure 4.** (**A**) Bliss independence assay in 4T1 cell line. n=3. Data are presented as mean±SEM. (**B**) Comparison of cell viability of sequential and concurrent treatment with 10 nM of ADC and $1x10^5$ MOI of cmLumiOpto in MDA-MB-231 cells. n=3. Data are presented as mean±SEM. (**C**) Analysis of the expression of apoptotic (cleaved caspase-3) or DNA damage (γ-H2AX) markers in sequential and concurrent treatments. (**D**) Seahorse assay to analyze mitochondrial function of MDA-MB-468 and 4T1 cells post treatment. n=4. Data are presented as mean±SEM.

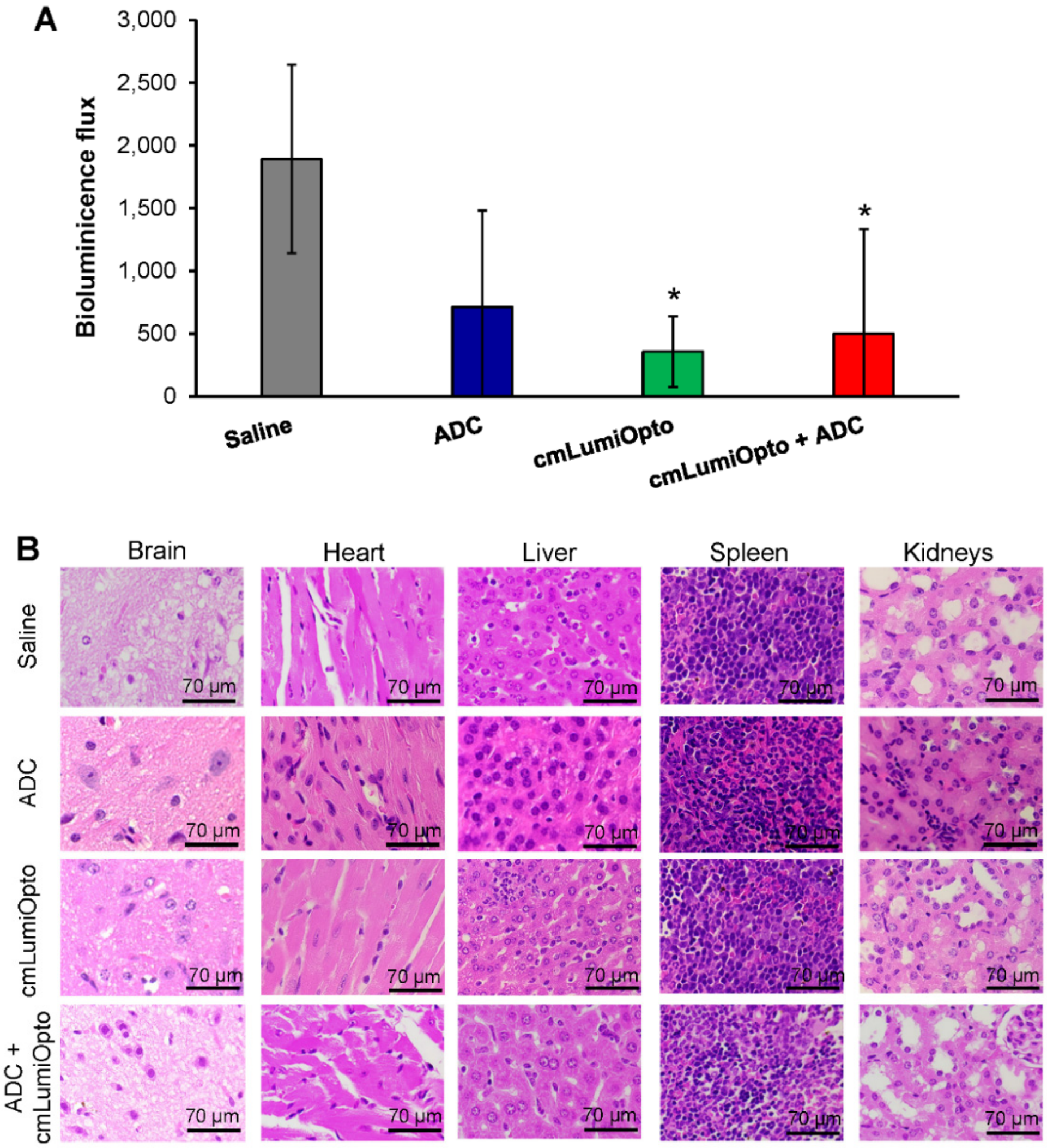

Figure S4. Potential toxicity evaluation in metastatic syngeneic models. Related to Figure 5. Mice were treated with saline (control), CD276 mAb-DM1 (8 mg/kg), cmLumiOpto (10x10^{10} ptc/kg), and combination of ADC/cmLumiOpto (same dose as monotherapy). n=5 mice/group. Data are presented as mean±SEM. (**A**) Tumor bioluminescence flux of mice in saline treatment groups. *P<0.005. (**B**) H&E staining of major organs harvested including brain, heart, liver, spleen and kidneys. Scale bar: 70 μm.

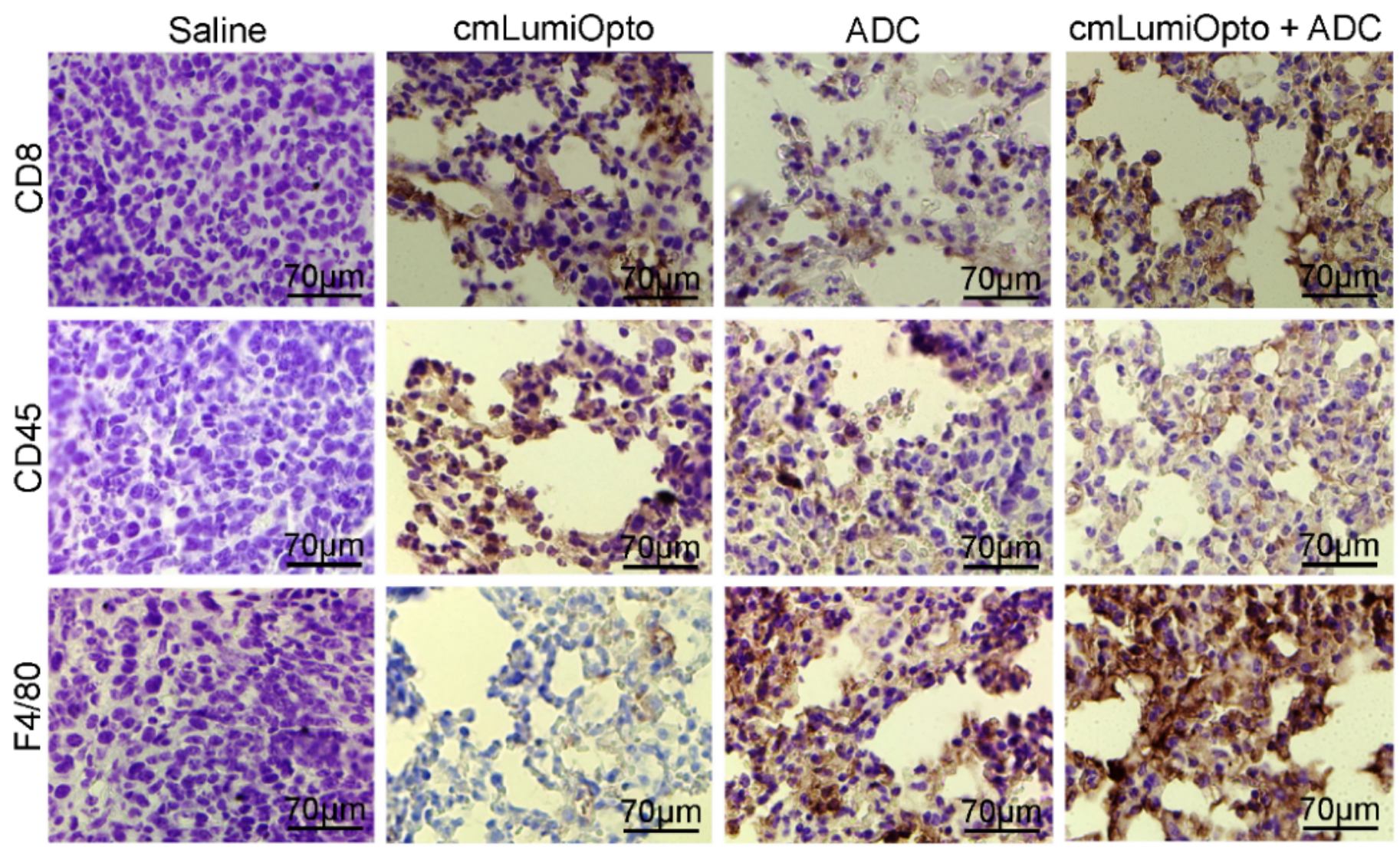

Figure S5. Immune activation evaluation in metastatic syngeneic models. Related to Figure 6. Mice were treated with saline (control), CD276 mAb-DM1 (8 mg/kg), cmLumiOpto ($10x10^{10}$ ptc/kg), and combination of ADC/cmLumiOpto (same dose as monotherapy). n=5 mice/group. IHC staining tumor slides with antibodies targeting CD8, CD45 and F4/80. Scale bar: 70 µm.
