# Supplementary material for: Combine antibody-drug conjugate with cmLumiOpto for triple-negative breast cancer treatment
Source: Cell Rep Med. 2026 Jul 24;7(8):102934. doi: 10.1016/j.xcrm.2026.102934 (PMC13522777; doi:10.1016/j.xcrm.2026.102934)
Supplement: Supplementary file 2 [file mmc2.pdf]

Cell Reports Medicine

Article

Combine antibody-drug conjugate with cmLumiOpto for triple-negative breast cancer treatment

Graphical abstract

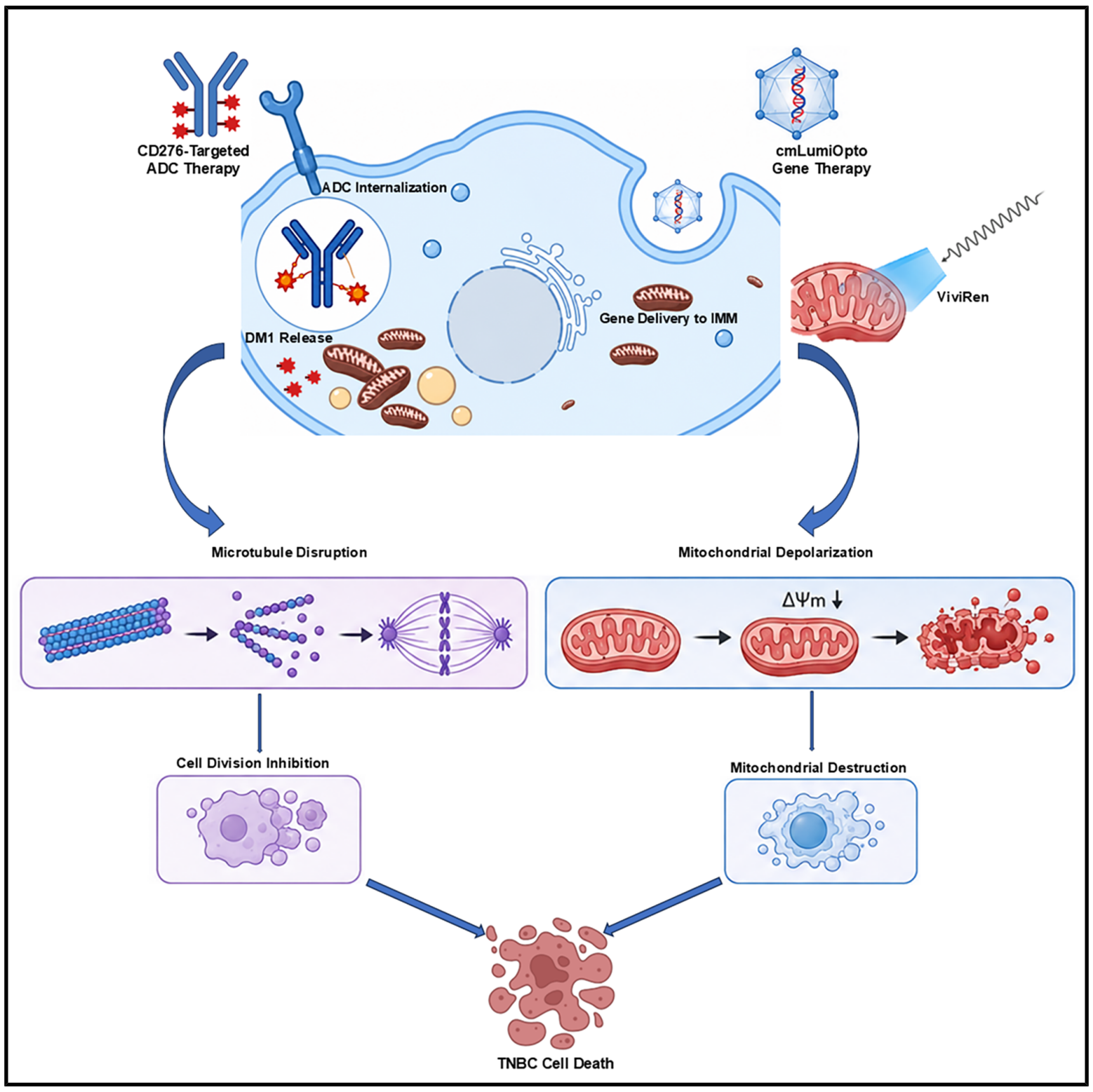

Authors

Tanvi Varadkar, Zhuoxin “Zora” Zhou, Yingnan Si, ..., Chandini Tsaliki, Lufang Zhou, Xiaoguang “Margaret” Liu

Correspondence

liu.482@osu.edu

In brief

Varadkar et al. show that combining a targeted antibody-drug conjugate with mitochondria-disrupting gene therapy shrinks tumors, halts metastasis, and activates the immune system in preclinical models with low toxicity, offering a promising therapeutic strategy for aggressive triple-negative breast cancer.

Highlights

- CD276-targeted ADC synergizes with cmLumiOpto to kill TNBC cells
- Combination suppresses TNBC metastasis in mouse models
- Inhibits tumor growth by 95%–100% in patient-derived xenografts
- Remodels the tumor microenvironment, boosts immunity, and shows low toxicity

Varadkar et al., 2026, Cell Reports Medicine 7, 102934
August 18, 2026 © 2026 The Author(s). Published by Elsevier Inc.
https://doi.org/10.1016/j.xcrm.2026.102934

CellPress

Cell Reports Medicine

CellPress
OPEN ACCESS

Article

Combine antibody-drug conjugate with cmLumiOpto for triple-negative breast cancer treatment

Tanvi Varadkar,[1,5] Zhuoxin "Zora" Zhou,[1,5] Yingnan Si,[1] Jiashuai Zhang,[2] Zhantao Du,[1] Anjana Gautam Khanal,[4] Srijita Chowdhury,[1] Chandini Tsaliki,[1] Lufang Zhou,[2,3,4] and Xiaoguang "Margaret" Liu[1,4,6,*]
[1]Department of Chemical and Biomolecular Engineering, The Ohio State University, Columbus, OH, USA
[2]Department of Biomedical Engineering, The Ohio State University, Columbus, OH, USA
[3]Department of Surgery, The Ohio State University, Columbus, OH, USA
[4]Comprehensive Cancer Center, The Ohio State University, Columbus, OH, USA
[5]These authors contributed equally
[6]Lead contact
*Correspondence: liu.482@osu.edu
https://doi.org/10.1016/j.xcrm.2026.102934

SUMMARY

Triple-negative breast cancer (TNBC) is an aggressive and chemotherapy-resistant subtype characterized by high metastatic potential and frequent recurrence, making monotherapies largely ineffective. This study evaluates a combination strategy utilizing a CD276 (B7-H3)-targeted antibody-drug conjugate (ADC) alongside mitochondria-targeted gene therapy. The anti-CD276 monoclonal antibody-mertansine conjugate substantially reduces tumor burden at a low dose. It exhibits strong synergy with the cmLumiOpto platform, enhancing cytotoxicity across three TNBC cell lines, impairing mitochondrial function, and elevating the tumoral immune response. Furthermore, the ADC/cmLumiOpto combination suppresses TNBC metastasis in mouse models and inhibits tumor growth by 95%–100% in patient-derived xenograft models. Mechanistic investigations reveal downregulated metastatic signaling, tumor microenvironment remodeling, increased apoptosis, and upregulated tumoral immunity. Histological and body weight analyses indicate minimal systemic toxicity. These findings highlight the translational potential of combining CD276-targeted ADC with mitochondrial gene therapy to treat aggressive TNBC.

INTRODUCTION

Triple-negative breast cancer (TNBC), defined as $HER2^-/ER^-/PR^-$, is clinically characterized by aggressive behavior, high metastatic potential, and considerable molecular heterogeneity.[1,2] Despite advances in oncology, the recurrence rates following initial treatment exceed 50%, and long-term survival remains poor.[3,4] Standard chemotherapy regimens often fail to achieve sustainable responses, particularly in recurrent or metastatic settings, due to acquired drug resistance and substantial systemic toxicity.[5,6]

Antibody-drug conjugates (ADCs), which link potent cytotoxic payloads to monoclonal antibodies (mAbs), have transformed treatment landscapes for multiple cancers.[7–9] Notable breast cancer therapies include HER2-targeted ado-trastuzumab emtansine and trastuzumab deruxtecan, as well as Trop-2-targeted sacituzumab govitecan and datopotamab deruxtecan.[10–15] Despite these achievements, many patients with TNBC do not benefit from ADC monotherapy due to the challenges of early metastases, high relapse rates,[16] intratumoral heterogeneity,[17] and drug resistance arising from compensatory mechanisms during prolonged treatment.[16,18,19]

CD276 (B7-H3), a type I transmembrane glycoprotein composed of two IgV-like and IgC2-like extracellular domains,[16] has emerged as an attractive therapeutic target in oncology. Our previous study demonstrated high CD276 expression in ~70% of TNBC patients (as well as in $HER2^+$ and ER^+/PR^+ subtypes)[20] and multiple non-small cell lung cancers.[21] Its expression is minimal in most normal tissues, including the brain, heart, lung, liver, pancreas, kidney, and normal breast, and remains low in steady-state fibroblasts and endothelial cells, though markedly it is upregulated in tumor-associated vasculature.[22,23] Recent clinical trials of CD276-targeted agents, including ifinatamab deruxtecan (NCT06292956 and NCT05280470) for small-cell lung cancer and vobramitamab duocarmazine (NCT05551117) for prostate cancer, have shown encouraging clinical benefits. Furthermore, CD276 functions as an immune checkpoint by suppressing effector cytokine secretion from natural killer (NK) and T cells.[24–26] Together, these attributes position CD276 as a promising target for both chemotherapy- and immunotherapy-based approaches. In previous studies, we developed anti-CD276 mAbs and constructed corresponding ADCs that significantly reduced tumor burden in TNBC and non-small cell lung cancer xenograft models.[20,21] Nevertheless, the therapeutic efficacy of CD276-targeted ADCs against metastatic TNBC, either as monotherapy or in combination regimens, warrants further exploration.

Emerging evidence, including our own prior research, underscores the central role of mitochondria in regulating cancer cell

© 2026 The Author(s). Published by Elsevier Inc.
This is an open access article under the CC BY license (http://creativecommons.org/licenses/by/4.0/).

proliferation, metabolism, and apoptosis, rendering it an attractive therapeutic target.[27,28] Irreversible depolarization of the inner mitochondrial membrane potential activates sustained apoptotic cascades, impairs energy metabolism, and culminates in cell death. Leveraging these insights, we developed a cancer mitochondria-targeted luminoptogenetic gene therapy, named cmLumiOpto, designed to selectively collapse mitochondria in malignant cells. This system employs a cancer-targeted delivery platform comprising mAb-tagged exosome-associated adeno-associated virus (mAb-Exo-AAV) vectors driven by a cancer-selective *cfos* promoter. Prior evaluation revealed that cmLumiOpto reduced tumor burden by >90% in orthotopic TNBC xenografts through induction of irreversible mitochondrial depolarization and persistent DNA damage.[27] A subsequent study demonstrated superior efficacy when cmLumiOpto was combined with poly(ADP-ribose) polymerase inhibitors compared with either gene therapy or chemotherapy alone, highlighting the promise of this combinatorial strategy.[29]

The objective of this study is to comprehensively evaluate the therapeutic potential of combining a CD276-targeted ADC with cmLumiOpto gene therapy in TNBC. Specifically, the TNBC surface binding, drug delivery and internalization, and tumor targeting of the CD276 mAb were first confirmed. *In vitro* anticancer cytotoxicity of mAb-mertansine (DM1), mAb-Exo-AAV carrying cmLumiOpto, and the ADC/cmLumiOpto combination were assessed. *In vivo* efficacy was rigorously examined in metastatic models and patient-derived xenograft (PDX) models, focusing on metastasis suppression and tumor growth inhibition. Synergistic mechanisms were elucidated through analyses of mitochondrial function, intratumoral cytokines, metastatic signaling, immune responses, and DNA damage. Collectively, these preclinical findings provide a robust foundation for combination therapies targeting aggressive TNBC cancer.

RESULTS

TNBC targeting of CD276 mAb

The anti-CD276 mAb was produced with a volumetric titer of ~80 mg/L (Figure 1A). Flow cytometry analysis revealed strong surface binding of Alexa Fluor 647 (AF647)-conjugated mAb, with binding rates of 99.5%, 99.6%, and 75.6% in human MDA-MB-231, MDA-MB-468, and murine 4T1 TNBC cell lines, respectively (Figure 1B). These data confirmed the cross-species reactivity of our CD276 mAb, supporting the translational relevance of murine models for predicting clinical outcomes.

TNBC targeting and intracellular payload delivery were assessed by live-cell confocal microscopy. The AF647-labeled mAb (red) robustly bound to the surface of GFP-expressing MDA-MB-468 cells (green) within 2 h of incubation and achieved complete cytoplasmic internalization by 24 h (Figure 1C), demonstrating efficient antigen-specific endocytosis suitable for payload delivery.

In vivo biodistribution was evaluated using *in vivo* imaging system (IVIS). The Cy5.5-conjugated anti-CD276 mAb selectively accumulated in both human MDA-MB-468-FLuc and murine 4T1-FLuc xenograft tumors, with a strong Cy5.5 signal (red) precisely co-localizing with the bioluminescent tumor signal (FLuc, green) (Figure 1D). Minimal fluorescence was detected in major organs (brain, heart, lung, spleen, liver, and kidney), underscoring high tumor selectivity. Collectively, these data established that the anti-CD276 mAb exhibited efficient tumor targeting in both human and murine TNBC models, providing a robust platform for targeted delivery of cytotoxic payloads and gene therapies.

Anti-TNBC efficacy of ADC

ADC construction and characterization

The CD276-DM1 ADC was generated via lysine-based conjugation using a sulfo-Succinimidyl 4-(N-maleimidomethyl)cyclohexane-1-carboxylate (SMCC) linker (Figure 2A). SDS-PAGE confirmed structural integrity post-conjugation (Figure 2B), while high-performance liquid chromatography (HPLC) analysis revealed >95% conjugation efficiency with expected heterogeneity (Figure 2C). Native mass spectrometry determined a drug-to-antibody ratio (DAR) distribution of 0 (4.58%), 1 (17.14%), 2 (25.02%), 3 (23.41%), 4 (16.09%), 5 (9.61%), and 6 (4.16%), yielding an average DAR of 2.76 in the lysine-based mAb-DM1 conjugation. These results confirm the successful synthesis of a high-quality mAb-DM1 ADC with consistent yield and conjugation efficiency.

In vitro *cytotoxicity*

Cytotoxic potency was evaluated in a 5-day IC_{50} assay across human (MDA-MB-231, MDA-MB-468) and murine (4T1) TNBC cell lines. Free DM1 exhibited IC_{50} values of 11.0, 20.7, and 9.1 nM in MDA-MB-231, MDA-MB-468, and 4T1 cells, respectively (Figure 2D). The CD276-DM1 ADC demonstrated comparable potency, with IC_{50} values of 31.7, 22.4, and 19.8 nM in the same cell lines (Figure 2E), confirming potent antigen-dependent cytotoxicity in TNBC cells.

In vivo *anti-tumor efficacy*

Anti-tumor activity was assessed in the syngeneic 4T1-FLuc orthotopic model. Administration of CD276-DM1 ADC (8 mg/kg) significantly suppressed tumor growth compared with saline or mAb (8 mg/kg) controls (Figure 3A), without an obvious change in body weight (Figure 3B). Histological analysis of tumors with H&E staining revealed extensive cell death in ADC-treated tumor (Figure 3C). Immunohistochemistry (IHC) analysis showed reduced Ki67 expression (proliferation marker) and elevated cleaved caspase-3 (apoptosis marker) (Figure 3D). Safety profiling detected no inflammation, apoptosis, necrosis, or organ damage in the major tissues (brain, heart, liver, kidney, lung, and spleen) from ADC-treated mice (Figure S1A). Complete blood count results remained unaltered by mAb treatment relative to the saline control (Figure S1B), indicating minimal systemic toxicity. The anti-TNBC efficacy was further validated in PDX models, where 8 mg/kg ADC completely abrogated tumor growth (Figure S1C). Together, these results demonstrated that the CD276-targeted ADC inhibited solid TNBC tumor progression potently and safely in preclinical models.

Synergism of ADC and cmLumiOpto

Construction and characterization of TNBC-targeted cmLumiOpto

Cancer mitochondria-depolarizing cmLumiOpto technology was established as previously described[27] (Figure S2A). In this study,

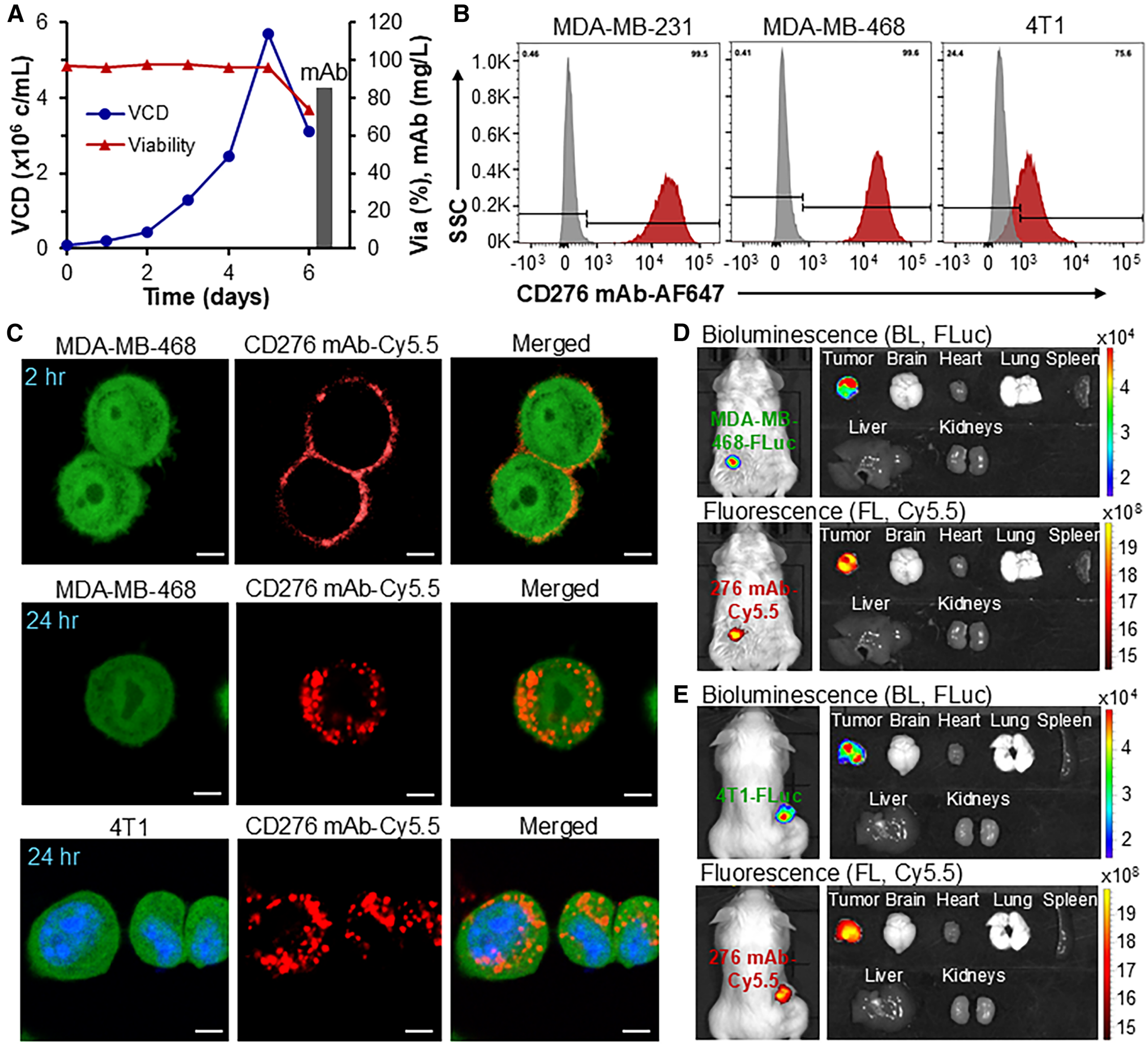

Figure 1. Anti-CD276 mAb production and evaluations

(A) Production of anti-CD276 mAb in a shaker flask at 37°C and 130 rpm.

(B) Flow cytometric analysis of TNBC (MDA-MB-231, MDA-MB-468, and 4T1) cell surface binding by CD276 mAb.

(C) TNBC targeting and internalization of CD276 mAb labeled with AF647 at 2 and 24 h. Scale bars: 10 μm.

(D and E) Live animal and *ex vivo* imaging to confirm the *in vivo* TNBC-specific targeting of CD276 mAb-Cy5.5 in MDA-MB-468-FLuc-xenografted NSG models (D) and in 4T1-FLuc-xenografted BALB/cJ models (E).

Images were captured at 24 h after tail vein injection.

AAV vectors encoding cmLumiOpto were encapsulated into exosomes, with the surface stabilized by conjugating DSPE-mPEG and further functionalized with an anti-CD276 mAb via a 1,2-Dimyristoyl-sn-glycero-3-phosphoethanolamine-polyethylene glycol-N-hydroxysuccinimide (DMPE-PEG-NHS) linker to enable TNBC-specific delivery (Figure S2B). High-yield production was achieved in a 2-L stirred-tank bioreactor, yielding approximately 1×10^{12} particles (ptcs)/mL with a homogeneous size distribution of 80–100 nm, as confirmed by NanoSight Pro analysis (Figure S2C). Effective cellular uptake and gene delivery were validated by confocal microscopy, demonstrating robust internalization of Cy5.5-labeled mAb-Exo-AAV (red) into GFP-expressing MDA-MB-468 cells (green) within 24 h after incubation (Figure S2D).

Synergism of ADC/cmLumiOpto combination

Synergistic cytotoxicity was evaluated in three TNBC cell lines (MDA-MB-231, MDA-MB-468, and 4T1). Cell viability was assessed at 48 h post-treatment and analyzed using the Bliss independence model, revealing synergistic effects across the majority of dose combinations (Figure 4A; Figure S3A). Robust synergy, defined by a combination index (CI) < 1, was observed in MDA-MB-231 cells at ADC concentrations >30 nM and cmLumiOpto levels $>1 \times 10^4$ MOI (12 of 25 conditions). In MDA-MB-468 cells, synergistic effects were even more prevalent, occurring in 19 of 25 tested conditions (Figure 4A). By contrast, synergism in 4T1 cells was relatively modest, with only 6 of 25 conditions yielding a CI < 1 (Figure S3A). Furthermore, we compared sequential (ADC followed by cmLumiOpto)

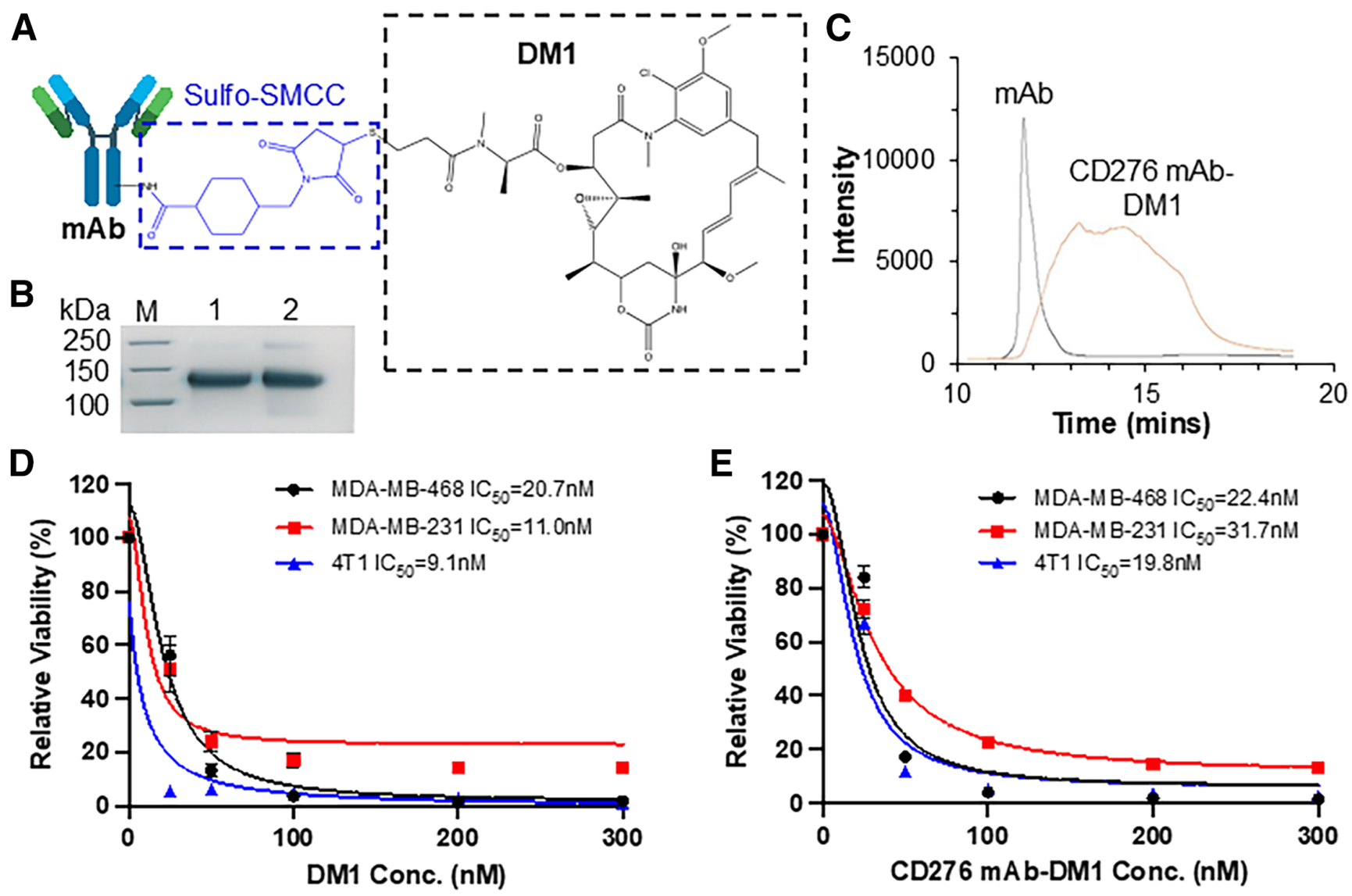

Figure 2. ADC construction, characterization, and *in vitro* cytotoxicity evaluation

(A) Structure of CD276 mAb-DM1 via sulfo-SMCC linker at lysine.

(B) SDS-PAGE of mAb and mAb-DM1. M, marker; 1, mAb; 2, ADC.

(C) Confirmation of ADC conjugation in HPLC equipped with a MAbPac HIC-Butyl column.

(D and E) Cytotoxicity of DM1 free drug (D) and ADC (E) in TNBC MDA-MB-231 cells (■), MDA-MB-468 cells (●), and 4T1 cells (▲). $n = 3$. Data are represented as mean ± SEM.

versus concurrent administration *in vitro*. Anti-cancer efficacy was comparable between the two regimens (Figure S3B), with no significant differences in the expression of apoptotic (cleaved caspase-3) or DNA damage (γ-H2AX) markers (Figure S3C).

The cytotoxicity was then assessed *in vitro* using two human TNBC cell lines (MDA-MB-468 and MDA-MB-231) with simultaneous treatment. While cmLumiOpto monotherapy at an MOI of 10,000 reduced cell viability to 50%–52% and 25 nM of ADC monotherapy reduced viability to 18%–30%, the combination further decreased viability to 8%–12% relative to saline (Figure 4B), indicating profound synergy in cancer cell killing. The mitochondrial bioenergetics were evaluated in MDA-MB-231, MDA-MB-468, and 4T1 cells using Seahorse XF analysis of oxygen consumption rate (OCR). Both monotherapies impaired mitochondrial function, but cmLumiOpto contributed the most mitochondrial damage, and the combination had a

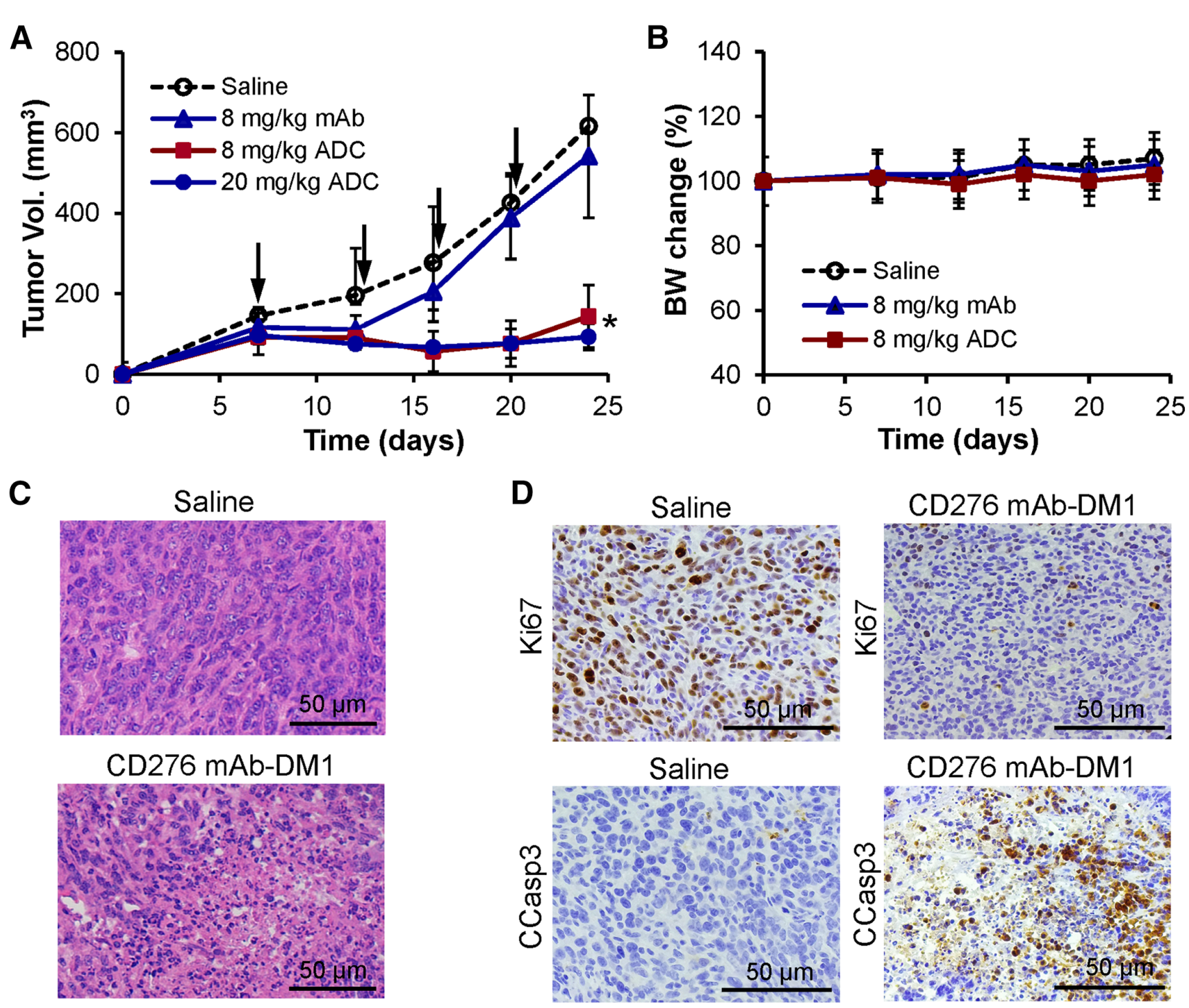

Figure 3. *In vivo* anti-TNBC efficacy of ADC in immunocompetent models

(A) Tumor volume profile. The mouse TNBC 4T1-FLuc line xenografted female BALB/cJ mice were treated with CD276 mAb-DM1, CD276 mAb, and saline (controls) following the schedule of Q4Dx4 as indicated by the black arrow. $n = 7$ mice/group. Data are presented as mean ± SEM. $^{*}p < 0.05$ vs. saline using ANOVA followed by Dunnett's *t* test.

(B) Changes in body weight.

(C) H&E staining of tumor tissues indicated low tumor intensity and cell death. Scale bars: 50 µm.

(D) IHC staining of tumor section demonstrated upregulation of apoptosis and inhibition of proliferation. Scale bars: 50 µm.

See also Figure S1.

CellPress
OPEN ACCESS

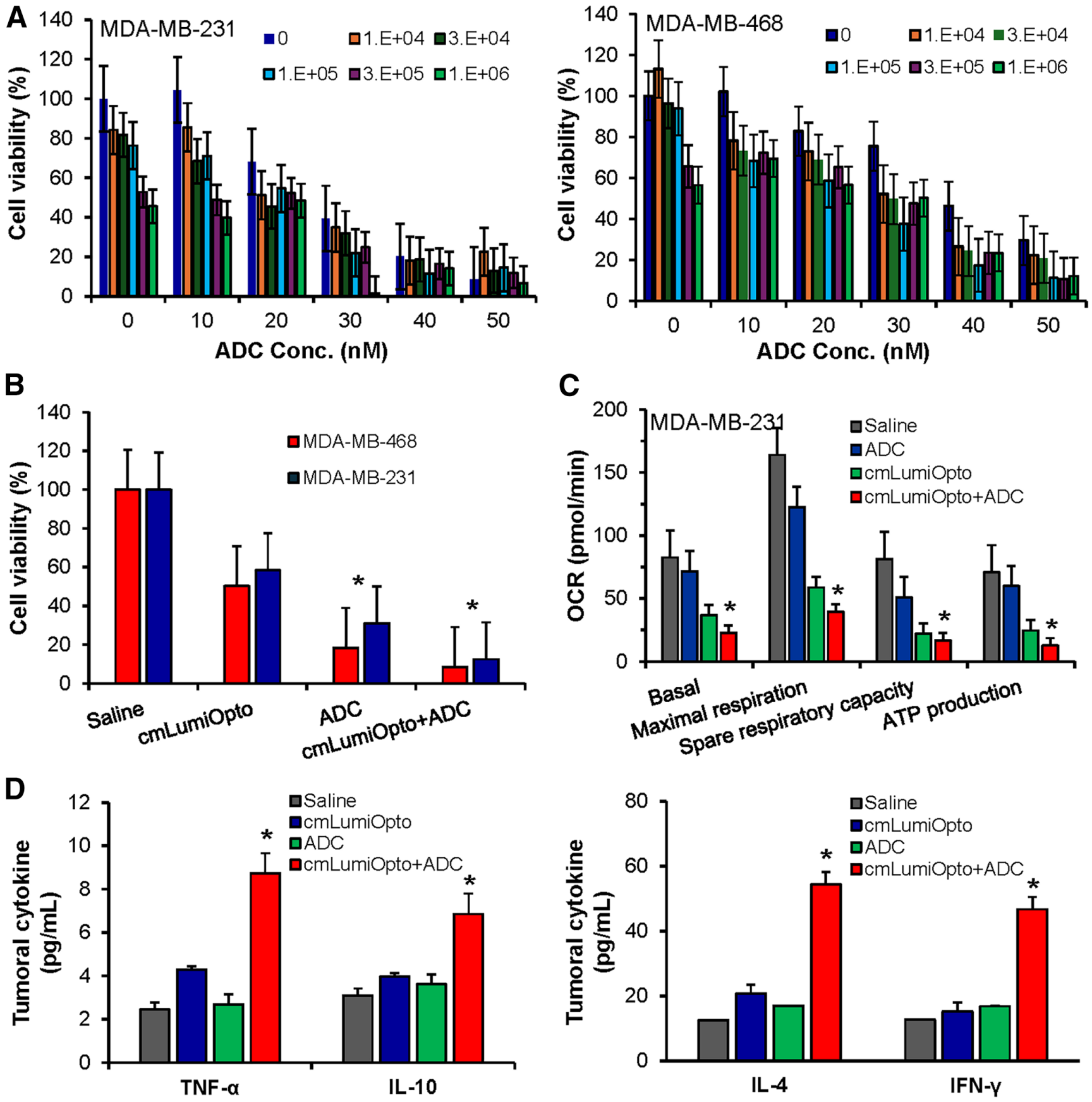

Figure 4. Synergism of ADC and cmLumiOpto

(A) Assessment of synergism of cmLumiOpto (1×10^4 MOI) and ADC (25 nM) using MDA-MB-231 and MDA-MB-468 cells. $n = 3$. Data are represented as mean ± SEM.

(B) Cytotoxic effects of saline, cmLumiOpto, ADC, and cmLumiOpto+ADC in MDA-MB-231 and MDA-MB-468 cells. $n = 3$. Data are represented as mean ± SEM.

(C) Seahorse assay to analyze oxygen consumption rate (mitochondrial function) of MDA-MB-231 cells after treatment. $n = 4$. Data are represented as mean ± SEM.

(D) Multiplex Luminex assay identified several enhanced cytokines in lung tissue carrying metastatic 4T1 tumor after ADC/cmLumiOpto treatment. $n = 4$. Data are represented as mean ± SEM. *$p < 0.05$.

See also Figures S2 and S3.

higher level of suppression (Figure 4C). Specifically, ADC/cmLumiOpto reduced basal respiration from ∼80 (saline) to ∼18 pmol/min, maximal respiration from ∼160 to ∼40 pmol/min, spare respiratory capacity from ∼80 to ∼20 pmol/min, and ATP-linked respiration from ∼60 to ∼15 pmol/min in MDA-MB-231 cells. Consistent Seahorse metabolic profiles were observed in MDA-MB-468 and 4T1 cells (Figure S3D), suggesting that the therapeutic efficacy is independent of specific cancer subtypes. To examine immunomodulatory effects, tumoral cytokine profiles in lung tissues carrying 4T1 metastases were quantified using a multiplex Luminex assay. The ADC/cmLumiOpto combination significantly elevated the levels of key cytokines compared with saline, including approximately 2-fold increases in TNF-α, IL-10, IL-4, and IFN-γ (Figure 4D). Collectively, these results demonstrated strong therapeutic synergy between the CD276-targeted ADC and cmLumiOpto, as indicated by cytotoxicity enhancement, profound mitochondrial dysfunction, and favorable remodeling of the TNBC immune microenvironment.

Metastatic reduction by ADC/cmLumiOpto combination

To evaluate the therapeutic efficacy of ADC/cmLumiOpto for the management of advanced TNBC, we employed immunocompetent mouse models carrying TNBC metastases in the lung. Treatments comprised saline (control), low-dose CD276-DM1 ADC (8 mg/kg), low-dose cmLumiOpto (1×10^{11} ptc/kg), or the ADC/cmLumiOpto combination (same dose). Metastatic progression was monitored by bioluminescent imaging (FLuc), with terminal lungs subjected to histopathological analysis of tumor burden, tissue structure, and cellular morphology.

In vivo IVIS imaging revealed that both monotherapies inhibited metastatic proliferation and disrupted niche establishment, but the combined therapy exerted markedly greater suppression of metastatic lesions (Figure 5A; Figure S4A). H&E staining was performed on lung sections collected from mice with strong FLuc signal. The saline group had extensive multifocal metastatic nodules occupying large portions of the lung lobes, with irregular borders and confluent growth, heavily circumscribed lesions, and preserved alveolar structure (Figure 5B). ADC and cmLumiOpto groups revealed comparable benefits, showing reduced nodule size and number compared with saline. Notably, ADC/cmLumiOpto resulted in a near-normal lung appearance, with only rare small nodules and one macroscopic lesion, demonstrating profound synergistic clearance of metastases. H&E staining of the brain, heart, liver, spleen, and kidney did not detect inflammation, apoptosis, necrosis, or organ damage, confirming minimal systemic toxicity (Figure S4B).

Higher magnification histopathological analysis of isolated metastatic foci provided cellular-level insights into the therapeutic effects on metastatic TNBC tumors (Figure 5C). The saline group showed viable and proliferative tumor cells, as indicated by the dense clusters of pleomorphic tumor cells with high nuclear-to-cytoplasmic ratios, prominent nucleoli, and mitosis. ADC monotherapy induced a strong treatment response, including substantial necrosis, cellular debris, and apoptotic features with residual viable tumor cells. cmLumiOpto caused similar disruptions of tumor integrity to those caused by the ADC, with fragmented nuclei and cytoplasmic vacuolization, suggesting programmed cell death. ADC/cmLumiOpto showed

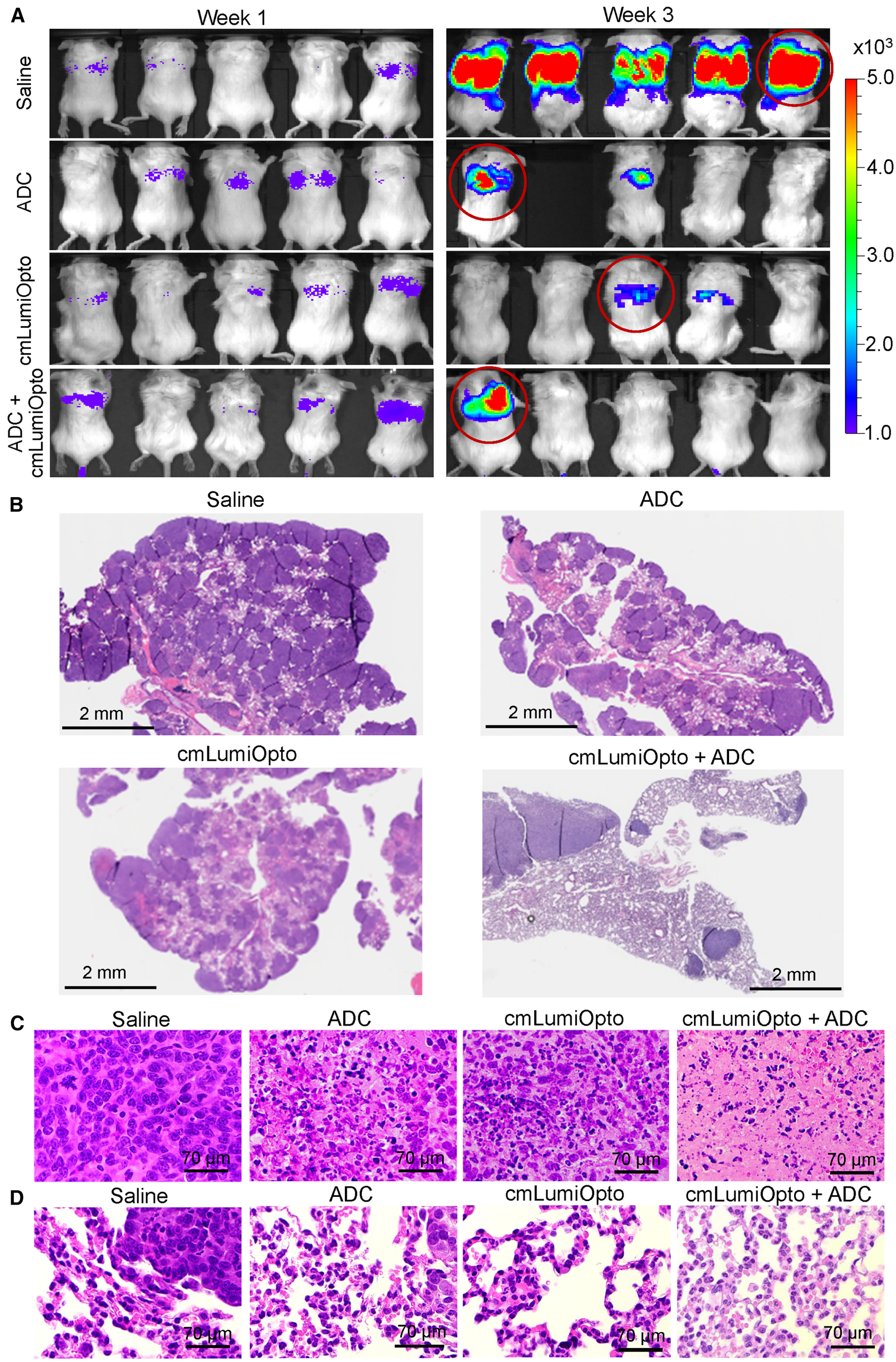

(legend on next page)

CellPress
OPEN ACCESS

predominant necrotic debris and fibrotic remnants with scant intact tumor cells, indicating potent synergistic eradication of metastatic TNBC foci in lung tissue. These images clearly revealed cytopathological alterations and enhanced tumor cell killing by the therapeutic combination.

Furthermore, we examined metastasis-involved lung tissue by focusing on the interaction between the tumor and the host (Figure 5D). The saline group showed that the invasive TNBC tumor front disrupted alveolar septa with lymphovascular invasion, which could promote further spread. ADC treatment attenuated invasion, with tumor cells showing pyknosis and being surrounded by increased stroma. cmLumiOpto reduced cellular atypia and improved tissue integrity. ADC/cmLumiOpto restored a near-normal lung architecture, with minimal residual tumor cells and resolved inflammation, reflecting comprehensive metastatic control. This result underscored the protective effects of the combinatorial treatment on host lung tissue.

In summary, while ADC and cmLumiOpto monotherapies partially suppressed TNBC lung metastasis, their combination achieved higher anti-metastatic efficacy, evidenced by significantly reduced bioluminescence, lower pathological burden, and extensive tumor cell death. These results substantiated the therapeutic potential of integrating CD276-targeted cytotoxic delivery with mitochondria-depolarizing gene therapy for managing advanced TNBC.

Mechanisms of action to reduce metastasis

To elucidate the mechanisms underlying metastatic suppression, bulk RNA sequencing was performed on lung tissues harboring TNBC metastases from the ADC/cmLumiOpto and saline groups. Differential gene expression analysis revealed the key pathway modulations, as presented in the enrichment bubble plots (Figure 6). ADC/cmLumiOpto treatment prominently downregulated the pro-metastatic and proliferative signaling pathways (Figure 6A). Notable enrichments included downregulation of WNT/β-catenin signaling, linked to epithelial-mesenchymal transition (EMT), invasion, angiogenesis, and colonization, as well as fibroblast growth factor receptor and interleukin-6 pathways, suggesting suppression of tumor progression and metastasis. Attenuation of transforming growth factor β (TGF-β) signaling further indicated a reduction of EMT and dissemination. Conversely, activation of the tumor-suppressive Hippo pathway was observed in response to treatment. Collectively, these changes suggest that ADC/cmLumiOpto disrupted the intrinsic drivers of tumor growth, invasion, and microenvironment remodeling that are critical for TNBC lung metastasis.

Immune-related pathways were significantly upregulated, highlighting the enhancement of anti-tumor immunity (Figure 6B). Enrichments in T cell-mediated responses, αβ T cell activation, and interleukin-12 signaling indicated upregulation of adaptive immunity, likely involving increased T cell infiltration, Th1 polarization, and IFN-γ production. These transcriptomic findings aligned with the elevated intratumoral cytokines (TNF-α, IFN-γ, IL-4, and IL-10) detected in the Luminex assay, indicating favorable remodeling of the immune microenvironment to combat metastatic cells.

Additional enrichments included disruption of the DNA damage response and epigenetic regulation (Figure 6C). Downregulation of p53-mediated pathways suggested impaired DNA repair and apoptosis evasion in tumor cells. Reduced acetyltransferase and histone lysine N-methyltransferase activities implied epigenetic reprogramming, potentially silencing pro-survival genes through chromatin compaction.

Furthermore, we investigated the correlation between the ADC/cmLumiOpto combination and the anti-tumor immune response. First, significant ADC release within the tumor microenvironment (TME) was observed in the treatment group (Figure 6D), suggesting that the CD276-targeting ADC may exert a potent bystander effect to eradicate heterogeneous TNBC cell populations. Second, we confirmed the induction of immunogenic cell death (ICD) through the detection of key markers. The cell surface translocation of calreticulin (CRT, red color), a critical "eat-me" signal, and the release of high-mobility group box 1 (HMGB1), a mediator of dendritic cell maturation, were observed. Confocal microscopy revealed strong colocalization of the membrane marker wheat germ agglutinin (WGA-FITC, green color) with the anti-CRT antibody-TRITC (red color) (Figure 6F), alongside the elevated HMGB1 levels (Figure 6E), validating that the treatment triggers both ICD and systemic immune activation. Third, the IHC analysis of tumor tissues with antibodies of CD8, CD45, and F4/80 demonstrated increased infiltration of $CD8^+$ T cells in the cmLumiOpto and combination groups, as well as activated $CD45^+$ T/NK cells and macrophages in the ADC-treated cohorts (Figure S5). Collectively, these findings showed that our integrated therapeutic strategy effectively upregulated tumoral immunity in TNBC.

In summary, ADC/cmLumiOpto elicited a multifaceted transcriptomic reprogramming in metastatic lungs, characterized by suppression of oncogenic/EMT signaling, activation of tumor-suppressive and immune pathways, and perturbation of DNA repair/epigenetic pathways. These coordinated effects underpinned the metastatic regression observed in preclinical TNBC models and provided a foundation for further functional validation.

Anti-TNBC efficacy in PDX models

To capture the heterogeneity of TNBC, we established PDX mouse models using JAX PDX donor mice (invasive ductal

Figure 5. *In vivo* anti-TNBC efficacy of combined therapy in metastatic syngeneic models

Mice were treated with saline (control), CD276 ADC (low dose of 8 mg/kg), cmLumiOpto (low dose of 10×10^{10} ptc/kg), and combination of ADC and cmLumiOpto (same dose as monotherapy). $n = 5$ mice/group.

(A) IVIS imaging of BALB/cJ mice carrying metastatic 4T1-FLuc.

(B) H&E staining of harvested lung tissue from the mouse with strongest bioluminescent signal. Scale bars: 2 mm.

(C) H&E staining of TNBC tumor spread in lung tissue. Scale bars: 70 μm.

(D) H&E staining of lung tissue bearing TNBC tumor. Scale bars: 70 μm.

See also Figure S5.

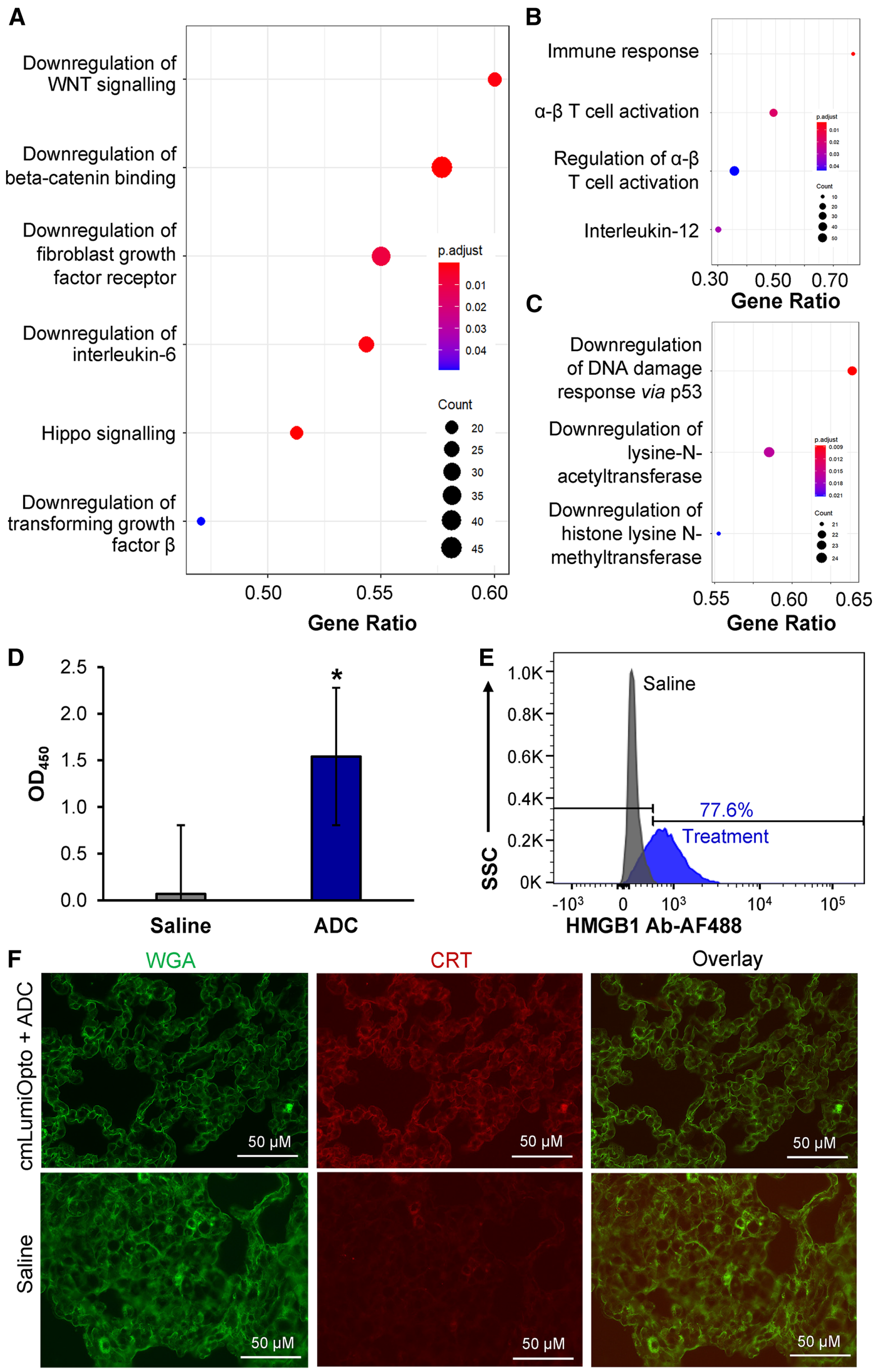

Figure 6. Mechanisms of action of ADC/cmLumiOpto

The lung tissues harvested from the same study as Figure 5 were used for bulk RNA sequencing. n = 4.

(A) Downregulation of metastasis signaling of Wnt, TGF-β, IL-6, and TGF after treatment.

(B) Upregulation of tumor immunity (T cells) and IL12.

(C) Downregulation of DNA damage response and histone lysine methylation. n = 4.

(D) Detection of ADC in the tumor microenvironment. n = 4. Data are represented as mean ± SEM.

(E) Tumoral HMGB1 analysis.

(F) Translation of ICD marker CRT.

See also Figure S5.

carcinoma, grade III, mesenchymal) to evaluate the therapeutic efficacy and safety profile of the ADC/cmLumiOpto combination (Figure 7). Tumor volume monitoring over 25 days revealed rapid progression in the saline control, increasing from ∼50 to 1,436 mm^3 (Figure 7A). In contrast, the combination therapy profoundly suppressed PDX growth, resulting in near-complete tumor stasis and significant tumor burden reduction. Histological analysis of harvested tumors at the end of treatment corroborated these findings (Figure 7B). Tumors from the saline group exhibited high density and proliferation, whereas tumors from the ADC/cmLumiOpto group displayed disrupted organization, reduced cell density, and extensive necrosis/apoptosis, consistent with the potent therapeutic effects observed in the metastatic models. Safety assessments further supported the clinical translatability of the therapy combination. Endpoint body weights were higher in the treatment group (28.7 g) compared with saline controls (20.9 g) (Figure 7C), indicating the absence of cachexia or systemic toxicity. Comprehensive H&E staining of major organs (brain, heart, liver, kidneys, lungs, and spleen) revealed no histopathological abnormalities, such as inflammation, necrosis, or structural damage, in either group (Figure 7D). Collectively, these results demonstrate that the ADC/cmLumiOpto combination elicits potent anti-tumor efficacy against heterogeneous TNBC in PDX models while exhibiting promising therapeutic safety, with minimal impact on animal health and organ integrity.

DISCUSSION

TNBC represents one of the most aggressive and heterogeneous subtypes of breast cancer, characterized by high rates of metastasis, recurrence, and dismal prognosis, with 5-year survival rates as low as 11% in advanced stages. Conventional monotherapies, including chemotherapy, ADC, and gene therapy, often yield limited durable responses, particularly in metastatic or recurrent patients, due to intrinsic resistance mechanisms and substantial toxicity. This study introduced an innovative multimodal strategy by integrating a CD276 mAb-delivered potent payload (mertansine) with a mitochondria-depolarizing gene therapy (cmLumiOpto). In two rigorous

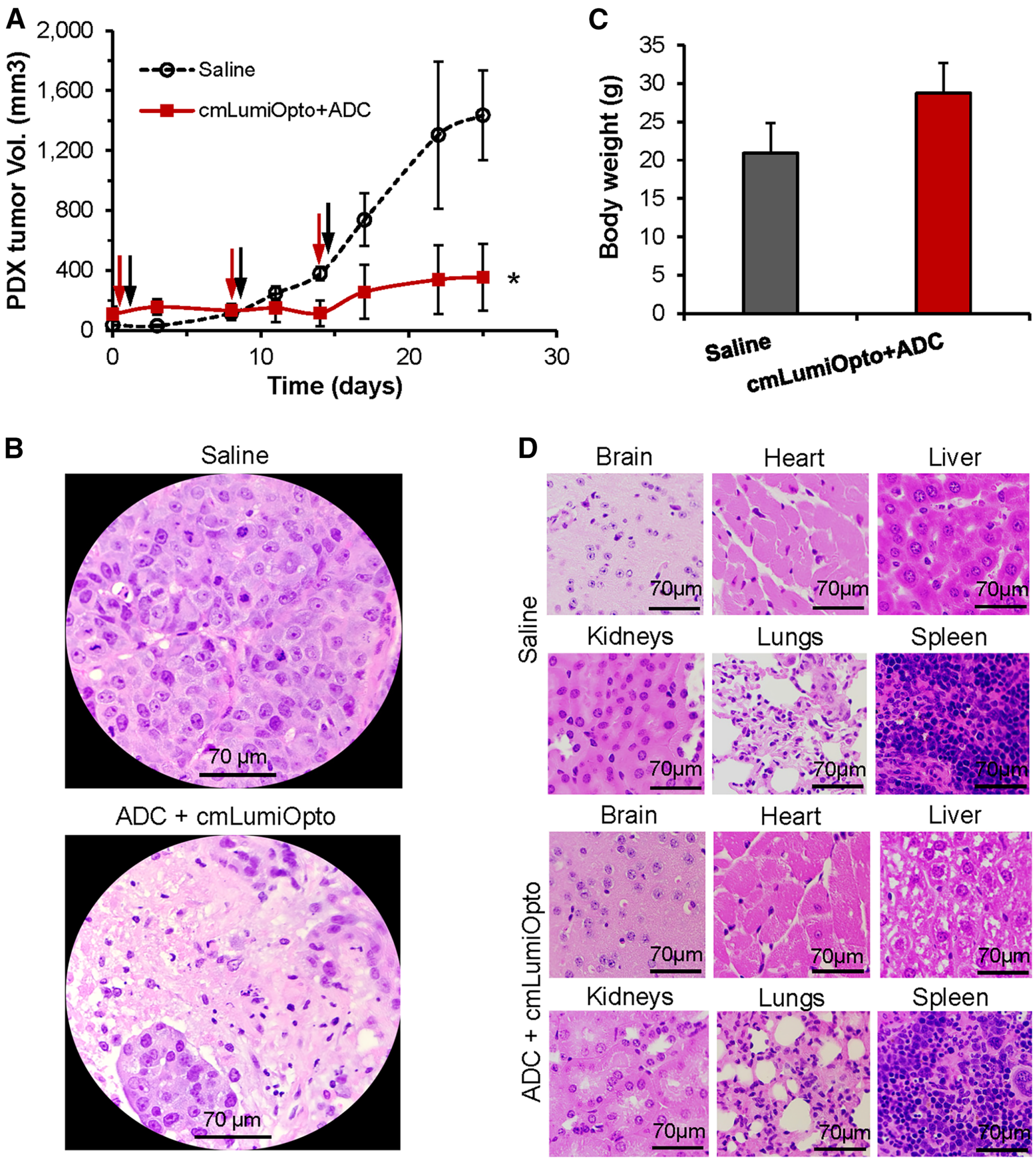

Figure 7. *In vivo* anti-TNBC efficacy of ADC/cmLumiOpto in PDX models

Mice were treated with a low dose of CD276 ADC (8 mg/kg)/cmLumiOpto (10×10^{10} ptc/kg) and saline (control). $n = 5$ mice/group.

(A) Tumor volume profiles. Data are represented as mean ± SEM.

(B) H&E staining of tumor after treatment. Scale bars: 70 μm.

(C) Body weight at endpoint.

(D) H&E staining of major organs including brain, heart, lungs, liver, spleen, and kidneys. Scale bars: 70 μm.

preclinical models recapitulating metastasis and tumor heterogeneity, the ADC/cmLumiOpto combination demonstrated remarkable therapeutic efficacy, positioning it as a pioneering approach to combat advanced TNBC.

The rationale for targeting CD276 is strongly supported by its overexpression in 60%–80% of diverse subtypes, coupled with minimal expression in most normal tissues, thereby providing a wide therapeutic index for selective tumor eradication.[20,21] Additionally, CD276 functions as an immune checkpoint inhibitor, suppressing NK and T cell effector cytokines, which further enhances the translational value of CD276-targeted strategies. Both ADC and cmLumiOpto delivered with mAb-Exo-AAV leveraged our CD276-targeting mAb, showing exceptional tumor selectivity, efficient payload/gene delivery, and cross-species reactivity.[20,21] *In vivo* biodistribution and toxicity assessments in this study confirmed precise tumor accumulation with negligible off-target effects in major organs, underscoring a favorable safety profile. The detected bystander effect of the ADC in the tumor microenvironment indicated its efficacy in treating heterogeneous TNBC. These preclinical observations align with emerging clinical data from CD276-directed ADCs in small-cell lung cancer and prostate cancer, where durable tumor regressions have been reported. Collectively, evidence from clinical trials,[24–26] our prior studies,[20,21] and this study supports CD276 as a compelling alternative to established targets like Trop-2 for TNBC and other cancers.

A central innovation of this study lies in the synergistic integration of CD276-DM1 ADC with cmLumiOpto gene therapy. Even at a low dose, ADC monotherapy substantially inhibited tumor progression in both syngeneic metastatic models and PDX models, while its combination with cmLumiOpto showed profound synergy. The dramatically impaired mitochondrial bioenergetics with reduced oxygen consumption rates were far beyond the rate seen with monotherapy. In metastatic models, this combination nearly eradicated lung metastases of TNBC, as revealed by bioluminescent imaging, whole-mount histopathological analysis, and high-magnification analyses revealing extensive tumor necrosis/apoptosis. Transcriptomic profiling of metastatic lungs elucidated the multifaceted mechanisms: downregulation of pro-metastatic signaling cascades, activation of tumor-suppressive Hippo signaling, and perturbation of DNA damage response and epigenetic regulation. Concomitantly, the robust upregulation of immune pathways correlated with enhancement of tumoral cytokines suggested the remodeling of TNBC microenvironmental immunity. The detection of multiple immune cell death markers (surface calreticulin and HMGB1) and infiltration of cytotoxic T cells, activated T and NK cells, and microphages in tumors after treatment indicated a strong correlation between immune activation and the combination of CD276-DM1 ADC with cmLumiOpto gene therapy. In heterogeneous PDX models, ADC/cmLumiOpto achieved near-complete abrogation of primary tumor growth without weight loss or histopathological organ damage, indicating minimal systemic toxicity. The synergistic anti-cancer mechanisms of the microtubule inhibitor delivered by the CD276-targeting mAb and the cancer mitochondrial damage induced by cmLumiOpto offer an effective therapeutic strategy to treat aggressive and heterogeneous CD276-positive TNBC.

These comprehensive anti-tumor and immunomodulatory effects underscore the complementary strengths of the approach, i.e., ADC-mediated direct cytotoxicity and bystander killing, augmented by cmLumiOpto-induced irreversible mitochondrial collapse, persistent DNA damage, and immune activation in the tumor microenvironment. Such synergistic mechanisms could effectively address the hallmarks of TNBC aggressiveness, such as metabolic reprogramming, immune evasion, and metastatic proficiency, while simultaneously mitigating dose-dependent toxicities. Comprehensive preclinical evaluation of dosing and regimens in future models will help identify the optimal combination strategy for these monotherapies and guide the design of first-in-human trials for advanced cancers.

In conclusion, this work established a transformative strategy for TNBC management by synergistically combining CD276-targeted ADC with mitochondria-directed cmLumiOpto gene therapy. Promising tumor control, metastatic reduction, immune activation, and minimal toxicity were achieved in evaluations using two preclinical models. If confirmed in future preclinical and clinical evaluations, ADC/cmLumiOpto could offer a promising therapeutic modality for high-grade TNBC and other CD276-positive malignancies.

Limitations of the study

Notwithstanding these encouraging results, several limitations require further investigation in future. First, the immunocompetent and heterogeneous models relying on mice may not fully capture the genomic, epigenetic, and microenvironmental complexity of human TNBC. Complementary *ex vivo* platforms such as patient-derived tumoroids or bioprinted 3D tissues and advanced immunocompetent models such as TNBC PDX-bearing humanized mouse could bridge this gap and provide additional mechanistic insights. Second, although low-dose regimens showed high therapeutic efficacy, optimization of dosage and treatment schedule will be essential for different stages of cancer. Third, the long-term therapeutic effects, such as potential toxicities, immunogenicity, and drug resistance or recurrence, remain to be thoroughly evaluated. Generation of Investigational New Drug (IND)-directed toxicology, pharmacology, and pharmacokinetic data using non-Good Laboratory Practice (GLP) murine and GLP non-human primate models, followed by a phase 1 clinical trial in CD276^{+} cancer patients, will be critical next steps.

RESOURCE AVAILABILITY

Lead contact

Further information and requests for resources and reagents should be directed to and will be fulfilled by the lead contact, Xiaoguang "Margaret" Liu (liu.482@osu.edu).

Materials availability

Most materials used in the study are commercially available. The NSG and BALB/cJ female mice were purchased from Jackson Laboratory. The anti-CD276 mAb, ADC, and mAb-Exo-AAV were generated in this study, which can be requested through university's technology office and intellectual property protection office.

Data and code availability

- All data generated or analyzed during this study are included in the main text or the supplemental materials of this article.
- The bulk RNA-seq data generated in this study are publicly available in Gene Expression Omnibus (GEO) with accession number GEO: GSE313892 at https://www.ncbi.nlm.nih.gov/geo/query/acc.cgi?acc=GSE313892. Raw and processed sequencing data are available through the GEO record.
- This paper does not report on the original custom code. All analyses were performed using publicly available software and R packages as described in the STAR Methods and key resources table.
- Any additional information required to reanalyze the data reported in this paper is available from lead contact upon request.

ACKNOWLEDGMENTS

The authors gratefully acknowledge the support provided by multiple core facilities at The Ohio State University, including the Comparative Pathology and Digital Imaging Shared Resource (CPDISR), Clinical and Translational Science Shared Resource (CTSSR), Genomics Shared Resource (GSR), Small Animal Imaging Shared Resource (SAISR), Flow Cytometry Shared Resource, Campus Microscopy & Imaging Facility (CMIF), and the Center for Electron Microscopy and Analysis. These resources contributed essential expertise and technical assistance in tissue sectioning, IVIS imaging, flow cytometry, nanoparticle tracking analysis, echocardiography, and transmission electron microscopy. We also acknowledge resources from the Campus Chemical Instrumentation Center Mass Spectrometry and Proteomics Facility and the OSU Comprehensive Cancer Center (OSUCCC) Proteomics Shared Resource (PSR). This facility is supported in part by funding from OSU's Enterprise for Research, Innovation and Knowledge and NCI grant P30 CA016058. The Q Exactive UHMR was supported by NIH P41 GM128577, with continuing technology optimization supported by RM1 GM149374. This work was supported by United States National Institutes of Health National Cancer Institute, NIH NCI) 1R01CA281980-01 (X.M.L.), R01CA262028 (X.M.L. and L.Z.), and R01HL156581 (L.Z.) and Department of Defense Breast Cancer Research Program (DOD BCRP) W81XWH2110066/67 (X.M.L. and L.Z.).

AUTHOR CONTRIBUTIONS

T.V. performed the experiments involving the development of mAb-Exo-AAV, nanoparticle tracking analyses, in addition to the corresponding studies involving combinatorial gene therapy (mAb-Exo-AAV) with CD276 ADC, cell-based assays, *in vitro* cytotoxicity, and animal studies. T.V. further took the lead in preparing the manuscript. Z.Z.Z. and Y.S. conducted the CD276 ADC developmental work, *in vitro* characterization, and corresponding animal models. J.Z. performed bulk RNA sequencing analysis and its corresponding statistical analysis. Z.D. performed the hematoxylin and eosin staining and immunofluorescent staining. S.C. and C.T. participated in Exo-AAV production, mAb conjugation, and characterization. A.G.K. supported animal studies including IVIS imaging. L.Z. provided funding support, contributed to experimental design, and prepared the manuscript. X.M.L. provided funding support, designed the whole study, and prepared and finalized the manuscript.

DECLARATION OF INTERESTS

A patent protecting the cmLumiOpto gene therapy and the mAb-Exo-AAV technology has been filed by The Ohio State University (PCT/US25/56581, "Mitochondrial optogenetics-based gene therapies and their methods of use"). The anti-CD276 mAb (PCT/US2024/037838) was licensed by BiFormyx Bio. X.M.L. serves as scientific co-founder and advisor for BiFormyx Bio and owns equity in this company.

STAR★METHODS

Detailed methods are provided in the online version of this paper and include the following:

- KEY RESOURCES TABLE
- EXPERIMENTAL MODEL AND STUDY PARTICIPATION DETAILS
  - Cell lines and culture media

- *In vivo* animal study
- METHOD DETAILS
  - Construction of ADC and cmLumiOpto
  - Characterizations of mAb, ADC and mAb-Exo-AAV
  - *In vitro* cytotoxicity assay
  - *In vivo* imaging system (IVIS)
  - Seahorse assay
  - Luminex assay
  - Bulk-RNA sequencing
  - Histology analysis
- QUANTIFICATION AND STATISTICAL ANALYSIS
  - Statistical analysis
- ADDITIONAL RESOURCES

SUPPLEMENTAL INFORMATION

Supplemental information can be found online at https://doi.org/10.1016/j.xcrm.2026.102934.

Received: December 20, 2025
Revised: May 7, 2026
Accepted: June 26, 2026
Published: July 24, 2026

REFERENCES

1. Tong, L., Yu, X., Wang, S., Chen, L., and Wu, Y. (2023). Research Progress on Molecular Subtyping and Modern Treatment of Triple-Negative Breast Cancer. Breast Cancer *15*, 647–658. https://doi.org/10.2147/BCTT.S426121.
2. Yin, L., Duan, J.J., Bian, X.W., and Yu, S.C. (2020). Triple-negative breast cancer molecular subtyping and treatment progress. Breast Cancer Res. *22*, 61. https://doi.org/10.1186/s13058-020-01296-5.
3. Hu, Y., Wang, C., Liang, H., Li, J., and Yang, Q. (2024). The treatment landscape of triple-negative breast cancer. Med. Oncol. *41*, 236. https://doi.org/10.1007/s12032-024-02456-9.
4. Leon-Ferre, R.A., and Goetz, M.P. (2023). Advances in systemic therapies for triple negative breast cancer. Br. Med. J. *381*, e071674. https://doi.org/10.1136/bmj-2022-071674.
5. Kim, C., Gao, R., Sei, E., Brandt, R., Hartman, J., Hatschek, T., Crosetto, N., Foukakis, T., and Navin, N.E. (2018). Chemoresistance Evolution in Triple-Negative Breast Cancer Delineated by Single-Cell Sequencing. Cell *173*, 879–893.e13. https://doi.org/10.1016/j.cell.2018.03.041.
6. Nedeljković, M., and Damjanović, A. (2019). Mechanisms of Chemotherapy Resistance in Triple-Negative Breast Cancer-How We Can Rise to the Challenge. Cells *8*, 957. https://doi.org/10.3390/cells8090957.
7. Zhou, X., Han, Y., Fang, Y., Ma, P., Zhou, J., Jiang, Y., Xing, S., Tang, Q., Hou, Y., Wang, S., and Li, N. (2025). Antibody-drug conjugates: Current challenges and innovative solutions for precision cancer therapy. Méd. Sur *6*, 100849. https://doi.org/10.1016/j.medj.2025.100849.
8. Mukherjee, A., and Bandyopadhyay, D. (2024). Targeted Therapy in Breast Cancer: Advantages and Advancements of Antibody-Drug Conjugates, a Type of Chemo-Biologic Hybrid Drugs. Cancers (Basel) *16*, 3517. https://doi.org/10.3390/cancers16203517.
9. Buyukgolcigezli, I., Tenekeci, A.K., and Sahin, I.H. (2025). Opportunities and Challenges in Antibody-Drug Conjugates for Cancer Therapy: A New Era for Cancer Treatment. Cancers (Basel) *17*, 958. https://doi.org/10.3390/cancers17060958.
10. Turshudzhyan, A. (2021). The role of ado-trastuzumab emtansine in current clinical practice. J. Oncol. Pharm. Pract. *27*, 150–155. https://doi.org/10.1177/1078155220951862.
11. Yaghoubi, N., Fatemi, B., Ahmadi, R., Metghalchi, Y., Shahrami, B., Vaezi, M., and Rezaei, S. (2025). Trastuzumab deruxtecan versus trastuzumab emtansine for human epidermal growth factor receptor 2 positive metastatic breast cancer: cost-effectiveness analysis from Iranian experience. BMC Health Serv. Res. *25*, 711. https://doi.org/10.1186/s12913-025-12876-6.
12. Shastry, M., Jacob, S., Rugo, H.S., and Hamilton, E. (2022). Antibody-drug conjugates targeting TROP-2: Clinical development in metastatic breast cancer. Breast *66*, 169–177. https://doi.org/10.1016/j.breast.2022.10.007.
13. Rossi, V., Turati, A., Rosato, A., and Carpanese, D. (2024). Sacituzumab govitecan in triple-negative breast cancer: from bench to bedside, and back. Front. Immunol. *15*, 1447280. https://doi.org/10.3389/fimmu.2024.1447280.
14. Orlandi, A. (2025). FDA and EMA approval of datopotamab-deruxtecan: A paradigm shift in breast cancer drug evaluation? Breast *84*, 104589. https://doi.org/10.1016/j.breast.2025.104589.
15. Mobashir, M., Tarique, H., Ahmad, S., Habib, I., Inam, A., Qamar, I., and Sajjad, W. (2025). Datopotamab deruxtecan: a new era in targeted therapy for metastatic breast cancer. Ann. Med. Surg. *87*, 6942–6943. https://doi.org/10.1097/MS9.0000000000003857.
16. Carey, L.A. (2011). Directed therapy of subtypes of triple-negative breast cancer. Oncologist *16*, 71–78. https://doi.org/10.1634/theoncologist.2011-S1-71.
17. Espinosa Fernandez, J.R., Eckhardt, B.L., Lee, J., Lim, B., Pearson, T., Seitz, R.S., Hout, D.R., Schweitzer, B.L., Nielsen, T.J., Lawrence, O.R., et al. (2020). Identification of triple-negative breast cancer cell lines classified under the same molecular subtype using different molecular characterization techniques: Implications for translational research. PLoS One *15*, e0231953. https://doi.org/10.1371/journal.pone.0231953.
18. Abelman, R.O., Wu, B., Spring, L.M., Ellisen, L.W., and Bardia, A. (2023). Mechanisms of Resistance to Antibody-Drug Conjugates. Cancers (Basel) *15*, 1278. https://doi.org/10.3390/cancers15041278.
19. Lev, S. (2020). Targeted therapy and drug resistance in triple-negative breast cancer: the EGFR axis. Biochem. Soc. Trans. *48*, 657–665. https://doi.org/10.1042/BST20191055.
20. Zhou, Z.Z., Si, Y., Zhang, J., Chen, K., George, A., Kim, S., Zhou, L., and Liu, X.M. (2024). A Dual-Payload Antibody-Drug Conjugate Targeting CD276/B7-H3 Elicits Cytotoxicity and Immune Activation in Triple-Negative Breast Cancer. Cancer Res. *84*, 3848–3863. https://doi.org/10.1158/0008-5472.CAN-23-4099.
21. Zhang, J., Zhou, Z.Z., Chen, K., Kim, S., Cho, I.S., Varadkar, T., Baker, H., Cho, J.H., Zhou, L., and Liu, X.M. (2023). A CD276-Targeted Antibody-Drug Conjugate to Treat Non-Small Lung Cancer (NSCLC). Cells *12*, 2393. https://doi.org/10.3390/cells12192393.
22. Hu, Z., Bachawal, S.V., Li, X., Wang, H., Wilson, K.E., Li, P., and Paulmurugan, R. (2022). Detection and Characterization of Sentinel Lymph Node by Ultrasound Molecular Imaging with B7-H3-Targeted Microbubbles in Orthotopic Breast Cancer Model in Mice. Mol. Imag. Biol. *24*, 333–340. https://doi.org/10.1007/s11307-021-01680-3.
23. Bachawal, S.V., Jensen, K.C., Wilson, K.E., Tian, L., Lutz, A.M., and Willmann, J.K. (2015). Breast Cancer Detection by B7-H3-Targeted Ultrasound Molecular Imaging. Cancer Res. *75*, 2501–2509. https://doi.org/10.1158/0008-5472.CAN-14-3361.
24. Getu, A.A., Tigabu, A., Zhou, M., Lu, J., Fodstad, Ø., and Tan, M. (2023). New frontiers in immune checkpoint B7-H3 (CD276) research and drug development. Mol. Cancer *22*, 43. https://doi.org/10.1186/s12943-023-01751-9.
25. Flem-Karlsen, K., Fodstad, Ø., and Nunes-Xavier, C.E. (2020). B7-H3 Immune Checkpoint Protein in Human Cancer. Curr. Med. Chem. *27*, 4062–4086. https://doi.org/10.2174/0929867326666190517115515.
26. Zhao, B., Li, H., Xia, Y., Wang, Y., Wang, Y., Shi, Y., Xing, H., Qu, T., Wang, Y., and Ma, W. (2022). Immune checkpoint of B7-H3 in cancer: from immunology to clinical immunotherapy. J. Hematol. Oncol. *15*, 153. https://doi.org/10.1186/s13045-022-01364-7.

27. Chen, K., Ernst, P., Sarkar, A., Kim, S., Si, Y., Varadkar, T., Ringel, M.D., Liu, X.M., and Zhou, L. (2024). mLumiOpto Is a Mitochondrial-Targeted Gene Therapy for Treating Cancer. Cancer Res. *84*, 4049–4065. https://doi.org/10.1158/0008-5472.CAN-24-0984.

28. Ernst, P., Xy, N., Qu, J., Chen, H., Goldberg, M., Darley-Usmar, V., Zhang, J., O'Rourke, B., Liu, X., and Zhou, L. (2019). Precisely control mitochondria with light to manipulate cell fate decision. Biophys J. *117*, 631–645.

29. Varadkar, T., Zhou, Z.Z., Zhang, J., Sarkar, A., Du, Z., Chowdhury, S., Lim, H., Zhou, L., and Liu, X.M. (2025). Combine mitochondrial-targeted gene therapy and chemotherapy to treat triple-negative breast cancer. J. Exp. Clin. Cancer Res. *44*, 328. https://doi.org/10.1186/s13046-025-03572-8.

30. Zhou, Z.Z., Ballard, D., Varadkar, T., Zhang, J., Du, Z., George, A., Krabacher, A., Yengo, R., Zhou, L., and Liu, X.M. (2025). Anti-lipolysis-stimulated lipoprotein receptor antibody-drug conjugate to treat triple-negative breast cancer. Front. Oncol. *15*, 1663743. https://doi.org/10.3389/fonc.2025.1663743.

31. Si, Y., Kim, S., Ou, J., Lu, Y., Ernst, P., Chen, K., Whitt, J., Carter, A., Bibb, J., Markert, J., et al. (2020). Anti-SSTR2 Antibody-drug Conjugate for Neuroendocrine Tumor Therapy. Cancer Gene Therapy. Cancer Gene Ther. *28*, 799–812.

32. Chen, K., Si, Y., Guan, J.S., Zhou, Z., Kim, S., Kim, T., Shan, L., Willey, C.D., Zhou, L., and Liu, X. (2022). Targeted Extracellular Vesicles Delivered Verrucarin A to Treat Glioblastoma. Biomedicines *10*, 130–144. https://doi.org/10.3390/biomedicines10010130.

33. Si, Y., Kim, S., Zhang, E., Tang, Y., Jaskula-Sztul, R., Markert, J.M., Chen, H., Zhou, L., and Liu, X.M. (2020). Targeted Exosomes for Drug Delivery: Biomanufacturing, Surface Tagging, and Validation. Biotechnol. J. *15*, 1900163–1900174. https://doi.org/10.1002/biot.201900163.

34. Si, Y., Chen, K., Ngo, H.G., Guan, J.S., Totoro, A., Zhou, Z., Kim, S., Kim, T., Zhou, L., and Liu, X. (2022). Targeted EV to Deliver Chemotherapy to Treat Triple-Negative Breast Cancers. Pharmaceutics *14*, 146. https://doi.org/10.3390/pharmaceutics14010146.

35. Si, Y., Guan, J., Xu, Y., Chen, K., Kim, S., Zhou, L., Jaskula-Sztul, R., and Liu, X.M. (2020). Dual-Targeted Extracellular Vesicles to Facilitate Combined Therapies for Neuroendocrine Cancer Treatment. Pharmaceutics *12*, 1079–1090.

36. Si, Y., Zhang, Y., Ngo, H.G., Guan, J.-S., Chen, K., Wang, Q., Singh, A.P., Xu, Y., Zhou, L., Yang, E.S., and Liu, X.M. (2021). Targeted liposomal chemotherapies to treat triple-negative breast cancer. Cancers *13*, 3749–3763.

37. Chen, K., Si, Y., Ou, J., Guan, J.S., Kim, S., Ernst, P., Zhang, Y., Zhou, L., Han, X., and Liu, X.M. (2021). Antibody-Drug Conjugate to Treat Meningiomas. Pharmaceuticals *14*, 427–439. https://doi.org/10.3390/ph14050427.

38. Si, Y., Xu, Y., Guan, J., Chen, K., Kim, S., Yang, E.S., Zhou, L., and Liu, X.M. (2021). Anti-EGFR antibody-drug conjugate for triple-negative breast cancer therapy. Eng. Life Sci. *21*, 37–44. https://doi.org/10.1002/elsc.202000027.

STAR★METHODS

KEY RESOURCES TABLE

REAGENT or RESOURCE	SOURCE	IDENTIFIER
**Antibodies**		
anti-CD276 mAb	In-house/Lab-produced	N/A
Anti-Cleaved Caspase-3 antibody	Abcam	Cat# ab214430; RRID: AB_2938798
Anti-Ki67 antibody	Abcam	Cat# ab16667; RRID: AB_302459
Anti-rabbit secondary antibody	Abcam	Cat# ab6721; RRID: AB_955447
**Bacterial and virus strains**		
Exo-AAV	In-house/Lab-produced	N/A
mAb-Exo-AAV	In-house/Lab-produced	N/A
**Biological samples**		
$CD276^+$ TNBC PDX tissue	The Jackson Laboratory	Cat# J000103917
**Chemicals, peptides, and recombinant proteins**		
DMEM medium	Gibco	Cat# 11995081
Fetal bovine serum (FBS)	Gibco	Cat# A5256701
Penicillin-Streptomycin-Glutamine	Gibco	Cat# 10378016
Glucose	Gibco	Cat# A2494001
L-Glutamine (200 mM)	Gibco	Cat# 25030164
GlutaMAX™ Supplement	Gibco	Cata# 35050061
Cell Boost #6	Cytiva	Cat# SH3086601
Mertansine (DM1)	MedChemExpress	Cat# HY-19792
Sulfo-SMCC	Fisher Scientific	Cat# PIA39268
Ammonium Sulfate $(NH_4)_2SO_4$	Fisher Scientific	Cat# A702-3
Na_3PO_4	Fisher Scientific	Cat# S472500
mPEG-DSPE	Nanocs	Cat# PG1-DS-2k
NucBlue Live ReadyProbes	Fisher Scientific	Cat# R37605
BacMam GFP Transduction Control	Fisher Scientific	Cat# B10383
DSPE-PEG-NHS	Nanocs	Cat# PG2DSNS2K
Cytiva HyClone™ Phosphate Buffered Saline (PBS)	Cytiva	Cat# SH30256FS
ViviRen™ *In Vivo* Renilla Luciferase Substrate	Promega	Cat# P1232
Ethanol	Fisher Scientific	Cat# A405P-4
Xylene	Fisher Scientific	**Cat#** X3P-1GAL
Hematoxylin	Fisher Scientific	Cat# NC9220898
Hydrochloric acid	Fisher Scientific	Cat# AA33257Q9
Ammonium hydroxide	Fisher Scientific	Cat# A669S-212
Eosin	Fisher Scientific	Cat# 6766007
Sodium peroxide (H_2O_2)	Fisher Scientific	Cat# S363-500
TBS, 20× Solution, pH 7.4	Fisher Scientific	Cat# AAJ75892K2
Triton X-100	Fisher Scientific	Cat# MTX15681
3,3′-diaminobenzidine	Fisher Scientific	Cat# AAH5400006
**Critical commercial assays**		
Alexa Fluor 647 antibody labeling kit	Fisher Scientific	**Cat#** A20173
sulfo-Cyanine5.5 Antibody Labeling Kit	Lumiprobe	Cat# 7321-10rxn
R&D Systems™ MTT Reagent	R&D Systems	**Cat#** 48-902-501

(*Continued on next page*)

Continued		
REAGENT or RESOURCE	SOURCE	IDENTIFIER
Procine Multiplex Luminex assay	R&D systems	Cat# LXSAMSM-13
Luminex MAGPIX	Luminex Corporate	Cat# MPX-PVER-K25
RNeasy Fibrous Tissue Mini Kit	Qiagen	Cat# 74704
Deposited data		
Bulk RNA-seq data	This paper	GEO accession number GEO: GSE313892
Experimental models: Cell lines		
Human TNBC cell line: MDA-MB-468	ATCC	Cat# HTB-132
Human TNBC cell line: MDA-MB-231	ATCC	Cat# HTB-26
Human TNBC cell line: MDA-MB-468-FLuc	GeneCopoeia	Cat# SL027
Human TNBC cell line: MDA-MB-231-FLuc	GenTarget	Cat# SC041
Mouse TNBC cell lines: 4T1	ATCC	Cat# CRL-2539
Mouse TNBC cell lines: 4T1-FLuc	ATCC	Cat# CRL-2539-LUC2
Viral Production Cell 2.0	Gibco	Cat# A49784
Experimental models: Organisms/strains		
NSG female mice	Jackson Lab	Cat# 005557
BALB/cJ female mice	Jackson Lab	Cat# 000651
Recombinant DNA		
AAV-DJ/8-cfos-NLuc-2A-ABCB-CoChR	Chen et al.[27]	N/A
pHelper plasmid	Cell Biolabs	Cat# VPK-400-DJ-8
pAAV-DJ/8 Rep-Cap Plasmid	Cell Biolabs	Cat# VPK-420-DJ-8
Software and algorithms		
FlowJo V11 software	Version 11	N/A
HISAT2	Version 2.1.0	N/A
edgeR	Version 4.2.1	N/A
clusterProfiler	Version 4.10.0	N/A
GraphPad Prism	Version 10.6.1	N/A
XPONENT software	N/A	N/A
Other		
125-mL shaker flaks	Fisher Scientific	**Cat#** PBV12-5
CellXpert C170 CO_2 Incubator	VWR	Cat# 6734010015
UNOsphere SUPrA affinity column	Bio-Rad	Cat# 12009321
NGC liquid chromatography	Bio-Rad	Cat# 7880003
High-performance liquid chromatography (HPLC)	Shimadzu	Cat# 220-91640-10
MAbPac HIC-Butyl Silica Hydrophobic Interaction HPLC Column, 5 m, 4.6 mm × 100 mm	Fisher Scientific	Cat# 088558
2-L stirred-tank bioreactor	Distek	Cat# 2022-8100
Cytiva Hiscreen Capto Core 400 column	Cytiva	Cat# 17372410
Amicon Ultra Centrifugal Filter, 100 kDa MWCO	MilliporeSigma	Cat# UFC9100
Amicon® Ultra Centrifugal Filter, 10 kDa MWCO	MilliporeSigma	Cat# UFC9010
BD LSRII flow cytometer	BD Biosciences	LSRII
Leica confocal microscope	Leica	Cat# LEIQU-0687858-A
Glass-bottom dish	Cellvis	**Cat#** NC0409658
NanoSight Pro	Malvern Panalytical	Cat# 10047258 (HBG5000)
Fisherbrand Surface Treated SterileTissue Culture Plates	Fisher Scientific	**Cat#** FB012930

(*Continued on next page*)

Continued

REAGENT or RESOURCE	SOURCE	IDENTIFIER
SpectraMax iD3 Plate Reader	BioTek	Cat# ID3-STD
Seahorse XF Analyzer	Agilent	Cat# S7850A
Cell Mito Stress Kit	Agilent	Cat# 103015-100
Agilent Seahorse XFe96/XF Pro FluxPak Mini	Agilent	Cat# 103793-100
16 G x 1-1/2 in. BD PrecisionGlide^{TM} Needle	BD	Cat# SKU305198

EXPERIMENTAL MODEL AND STUDY PARTICIPATION DETAILS

Cell lines and culture media

Human female TNBC cell lines MDA-MB-468 (ATCC, Cat# HTB-132, RRID:CVCL_0419, Manassas, VA, USA), MDA-MB-231 (ATCC, Cat# HTB-26, RRID:CVCL_0062), MDA-MB-468-FLuc (GeneCopoeia, Cat# SL027, RRID:CVCL_ZJ00, Rockville, MD, USA), and MDA-MB-231-FLuc (GenTarget, Cat# SC041, RRID:CVCL_YZ80, San Diego, CA, USA) were maintained in DMEM medium with 10% fetal bovine serum (FBS, v/v) and 1% Pen/Strep (v/v). The mouse female TNBC cell lines 4T1 (ATCC, Cat# CRL-2539, RRID:CVCL_0125) and 4T1-FLuc (ATCC, Cat# CRL-2539-LUC2, RRID:CVCL_5I85) were cultivated in RPMI-1640 medium with 10% FBS and 1% P/S. The CD276 mAb producing female mouse cells were maintained in serum free medium (SFM) with 6 g/L glucose and 6 mM L-glutamine in 125-mL shaker flaks at 37°C and Agtation 130 rpm. Viral Production Cell 2.0 (female, Gibco, Cat# A49784, RRID: RRID:CVCL_0045, Grand Island, NY) were cultivated in viral production medium with 4 mM GlutaMAX in shaker flask at an agitation speed of 120 rpm. The female TNBC PDX lines carried in donor mice (Jackson Lab, Cat# J000103917, Bar Harbor, ME, USA) were freshly frozen and stored in liquid nitrogen. All cell lines were maintained at 37°C and 5% CO_2 in a humidified incubator (Eppendorf, Enfield, CT, USA). All culture media and supplements were purchased from Fisher Scientific (Waltham, MA, USA) or Gibco, unless otherwise specified. All these cell lines were authenticated with genetics profiling for polymorphic short tandem repeat analysis at Genomics Core Facility. The in-house mycoplasma test of a large cell bank (>100 vials) was performed with PCR analysis of 6S rRNA genes on November 21, 2022. The cell culture period of the tested cell bank was controlled within 2–3 weeks.

In vivo animal study

Cell line-derived xenograft models and in vivo *treatment.* The 4T1-FLuc cells (5×10^6) were s.c. injected to 6-week BALB/cJ female mice (Jackson Lab, Cat# 000651, PRID: IMSR_JAX:000651). When tumor volume reached 100–150 mm^3, mice were randomized into four groups and injected with saline (control), 8 mg/kg of mAb (control), 8 mg/kg of ADC and 20 mg/kg of ADC via tail vein injection following schedule of Q4Dx4 (n = 7/group). Tumor volume was measured via vernier calipers and body weight was monitored weekly. When the early removal criteria (i.e., ulceration >2 mm) were met, mice were scarified to harvest tumor tissue and major organs (brain, heart, lung, kidneys, liver, and spleen) for histological analysis.

Metastatic models and in vivo *treatment.* About 2×10^6 mouse TNBC 4T1-FLuc cells were intravenously (i.v.) injected into 7-week-old BALB/cJ female mice through tail vein. IVIS imaging was carried out twice a week to monitor FLuc signal, which indicated the metastatic progression *in vivo*. Once obvious metastasis was observed, mice (n = 5/group) were treated with saline (control), CD276 mAb-DM1 (8 mg/kg), cmLumiOpto (10×10^{10} particles/kg of mAb-Exo-AAV and 2 mg/kg of ViviRen), and the combination of ADC and cmLumiOpto (same dose). IVIS imaging was performed to monitor. TNBC metastasis weekly. When body weight decreased by 20%, mice were sacrificed to harvest brain, heart, lungs, liver, spleen, and kidneys for multiple post-treatment analysis suchas mRNA sequencing, cytokine titration and histology.

Patient-derived xenograft (PDX) models and in vivo *treatment.* The female $CD276^+$ TNBC patient-derived xenograft (PDX) model was sourced from The Jackson Laboratory (Cat# J000103917). This model was established from a primary breast tumor diagnosed as Grade III invasive ductal carcinoma (AJCC stage IA). IHC analysis confirmed the presence of mesenchymal features, as evidenced by positive vimentin staining. The PDX mouse models were generated following our published procedure.[20,29,30] Briefly, the frozen $CD276^+$ TNBC PDX tissues from Jackson Lab were xenografted into 7-week-old NSG female mice (NOD.Cg-Prkdc^{scid} Il2rg^{tm1Wjl}/SzJ, Jackson Lab, Cat# 005557, PRID: IMSR_JAX:005557) by s.c. injecting a small fragment (~1 mm^3) using 6G needle (BD, Franklin Lakes, NJ, USA). When tumor volume reached 100 mm^3, mice were treated with saline (control) and ADC/cmLumiOpto combination at dose of 8 mg/kg/10×10^{10} particles/kg via i.v. injection following schedule of Q4Dx4 (n = 5). The endpoint was tumor volume in control group >1,000 mm^3. Major organs and tumor tissue were harvested for further histological analysis.

Ethics approval and consent to participate. All animal studies were conducted according to the Institutional Animal Care and Use Committee (IACUC) Protocol IACUC-2022A00000029 and 2023A00000039, which was approved by the Institutional Biosafety Committee at the Ohio State University. As breast cancer occurs in women in most cases, we used female mice only for TNBC xenograft models.

METHOD DETAILS

Construction of ADC and cmLumiOpto

CD276 mAb-DM1. The anti-CD276 mAb was produced from suspension culture supplemented with 3.5 g/L of Cell Boost #6 in shaker flask at 130 rpm and 37°C. mAb was purified using UNOsphere SUPrA affinity chromatography cartridge with NGC liquid chromatography system (Bio-Rad, Hercules, CA, USA) following our previously reported procedure.[20,21,30,31] The mAb-DM1 ADC was constructed following our established platform.[20,21,30,31] Specifically, the purified mAb was desalted and concentrated to 10 mg/mL using 10 kDa MWCO Amicon PES centrifuge filter unit (MilliporeSigma, Burlington, MA, USA). Then mAb, 10 mg/mL of Sulfo-SMCC linker, and 10 mM of DM1 were mixed with a molar ratio of 1:14:18.2 and incubated at 37°C for 1h. The synthesized ADC was purified using UNOsphere SUPrA protein A column and characterized with high-performance liquid chromatography (HPLC) (Shimadzu, Columbia, MD, USA). The HPLC equipped with a MAbPac hydrophobicity interaction chromatography (HIC)-butyl column (5 μm, 4.6 × 100 mm) was loaded and eluted with phase A of 2 M $(NH_4)_2SO_4$ and 100 mM Na_3PO_4 (pH 7.0) and phase B of 100 mM Na_3PO_4 (pH 7.0), respectively.

mAb-Exo-AAV carrying cmLumiOpto. Exo-AAV was produced from Viral Production Cell via transient transfection with three plasmids in a 2-L stirred-tank bioreactor (Distek, North Brunswick, NJ, USA) at 37°C, pH 7.0, 210 rpm and DO 40% as reported before.[27,29,32,33] Then, the spent medium containing Exo-AAV was harvested from bioreactors when viability dropped to 60–80%. Purification was performed using liquid chromatography equipped with 5-mL Cytiva Hiscreen Capto Core 400 column (Cytivia, Marlborough, MA, USA), followed with Amicon 100 kDa MWCO regenerated cellulose filter for buffer exchange and concentration. Finally, the purified Exo-AAV was modified using mPEG-DSPE to improve plasma stability and conjugated with our anti-CD276 mAb via a DSPE-PEG-NHS linker following previously published protocols.[27,29,32,34–36]

Characterizations of mAb, ADC and mAb-Exo-AAV

Flow cytometry analysis

The surface binding rates of CD276 mAb to three TNBC cell lines (MDA-MB-231, MDA-MB-468, 4T1) were tested using flow cytometry. As reported before,[20,21,30,31] 1×10^6 TNBC cells were stained with 5 μg of CD276 mAb-AF647 at 37°C for 30 min, washed with PBS for three times, and analyzed using BD LSRII flow cytometer (BD Biosciences, San Jose, CA, USA) with FlowJo V11 software for data processing with gating set of 0.5% fluorescent population of control cells.

Live-cell confocal microscopy

Live-cell confocal imaging was performed to validate the drug delivery ability of CD276 mAb. Briefly, the human MDA-MB-468 cells and mouse 4T1 cells were seeded in glass bottom μ-slide with a density of 5×10^4 cells/mL in 100 μL medium per well. BacMam GFP Transduction Control and NucBlue Live ReadyProbes were used to stain cytoplasm and nucleus, respectively. Then the AF647-mAb was added to cells with final concentration of 1 μg mAb/well. Live-cell images were captured at 2 h and 24 h after adding mAb using a Nikon A1R-HD25 confocal microscope with a high-speed resonance scanner (Nikon USA, Melville, NY, USA). The internalization of cmLumiOpto, delivered with mAb-Exo-AAV, was also analyzed using confocal microscopy. About 1×10^4 GFP-labelled MDA-MB-468 cells were seeded in 15-mm glass-bottom dish (Cellvis, Mountain View, CA, USA) and incubated overnight, followed with adding 1×10^9 particles of mAb-Exo-AAV-labelled with cyanine-5.5 fluorescence dye (Lumiprobe, Hunt Valley, MD, USA). After 24 h of incubation, live-cell images were acquired using Leica confocal microscope (Leica Camera AG, Teaneck, NJ, USA) with 640 nm and 488 nm lasers for cyanine-5.5 and GFP fluorescence detection, respectively.

Nanoparticle tracking analysis

mAb-Exo-AAV samples were diluted in PBS with factor of 1:10,000, and injected into microfluidics device of NanoSight Pro (Malvern Panalytical, Malvern, UK) at pump speed of 3 μL/min. The particles were imaged at setpoints of 5 captures per sample, 60 s per capture and 6 cameras. The size distribution and particle concentration of mAb-Exo-AAV were analyzed.

In vitro cytotoxicity assay

Anti-CD276 mAb-DM1

The human and mouse TNBC cells were seeded at densities of 1×10^4, 1×10^3 and 500 cells/mL for MDA-MB-468, MDA-MB-231 and 4T1, respectively, in 96-well plate. ADC was added into each well at dosages of 0, 2, 4, 10, 20, 50, 100, 200, and 300 nM, and incubated for five days at 37°C and 5% CO_2. $n = 3$. The relative cell viabilities were analyzed using MTT Cell Proliferation Assay kit.[34,37,38] The absorbance of each well, including the blank well with medium only, was measured at 570 nm in a microplate plate reader (BioTek, Winooski, VT, USA).

ADC/cmLumiOpto

TNBC cells were seeded at 5×10^4, 1.5×10^4 and 2.5×10^3 cells/mL for MDA-MB-468, MDA-MB-231 and 4T1, respectively, in 96-well plate. TNBC cells were treated with mAb-DM1 (25 nM, thrice), cmLumiOpto (mAb-Exo-AAV MOI of 10,000, once; 30 μM of ViviRen for three-day induction), and combined ADC/cmLumiOpto (same dose). $n = 3$. The relative viability was measured using the MTT Cell Proliferation Assay kit after five-day treatment. The absorbance was measured at 570 nm using a microplate plate reader (BioTek).

In vivo imaging system (IVIS)

About 5×10^6 of human TNBC MDA-MB-468-FLuc cells were subcutaneously (s.c.) injected into NSG (NOD.Cg-Prkdc<scid> Il2rg<tm1Wjl>/SzJ, Jackson Lab, Cat# 005557, Bar Harbor, ME, USA) female mice to generate immunocompromised models.

The 5 × 10^6 of mouse TNBC 4T1-FLuc cells were s.c. injected into BALB/cJ female mice to establish immunocompetent models. When tumor volume reached 200 mm^3, 40 μg of CD276 mAb labeled with cyanine-5.5 was injected into mice via tail vein. The live-animal IVIS images were captured at 24 h post injection. Major organs and tumors were harvested for *ex vivo* IVIS imaging.

Seahorse assay

TNBC MDA-MB-231 were seeded at a density of 5,000 cells/well in 96-well plate and incubated overnight. Then saline (control), cmLumiOpto (MOI 10,000 of mAb-Exo-AAV and 30 μM of ViviRen), CD276 mAb-DM1 (15 nM daily for four days), and combined mAb-DM1 and cmLumiOpto. $n = 4$. Mitochondrial activity was assessed after five-day treatment using a Seahorse XF Analyzer (Agilent, Santa Clara, CA, USA) with Cell Mito Stress Kit (Agilent).

Luminex assay

Procine Multiplex Luminex assay (R&D systems, Minneapolis, MN) was utilized to titrate cytokines and chemokines accumulation after treatment with saline, ADC, cmLumiOpto, and ADC/cmLumiOpto in 4T1 tumor microenvironment. The intracellular proteins were extracted from tumor tissues for Plex-13 assay. $n = 4$. Fluorescence intensity (MFI) was detected using Luminex MAGPIX (Luminex Corporate, Austin, TX, USA) and the raw data were analyzed using XPONENT software following manufacture instruction.

Bulk-RNA sequencing

Lung tissues harboring TNBC metastases were enzymatically dissociated, and total RNA was isolated using the RNeasy Fibrous Tissue Mini Kit (Qiagen, Germantown, MD, USA). cDNA libraries were prepared and sequenced by Novogene America (Sacramento, CA, USA) using Illumina HiSeq X Ten system. Bulk RNA-seq was performed using lung tissue samples from saline control and ADC/cmLumiOpto combination-treated mice, with $n = 4$ biological samples per group. Raw sequencing reads were aligned to the mouse reference genome (GRCm38/mm10) using HISAT2 (v2.1.0). Differential gene expression analysis between treatment and saline control groups was performed using edgeR (v4.2.1) in R. P values were adjusted for multiple comparisons using the Benjamini-Hochberg method, and genes with a false discovery rate (FDR) < 0.05 were considered significantly differentially expressed. Gene Ontology (GO) enrichment analysis was conducted using the clusterProfiler package (v4.10.0) in R and GO terms with adjusted $p < 0.05$ were considered significantly enriched. The bulk RNA-seq data generated in this study have been deposited in the Gene Expression Omnibus under accession number GEO: GSE313892.

Histology analysis

Hematoxylin and Eosin (H&E) staining

Paraffin-embedded tissue sections (~4 μm) were dewaxed in xylene and rehydrated through a graded ethanol series (100%–50%) to deionized water. Then slides were stained with hematoxylin, differentiated in 1% hydrochloric acid and 70% alcohol, blued in 1% ammonium hydroxide for 30 s, counterstained with eosin, dehydrated in 95% and 100% ethanol, cleared in xylene, and mounted.[20,21,27,30,32]

Immunohistochemistry (IHC) staining

The rehydrated sections were subjected to heat-induced epitope retrieval in 0.01 M sodium H_2O_2 for 15 min, and nonspecific binding was blocked with 5% normal goat serum in TBST (0.05 M Tris, 0.15 M NaCl, 0.1% Triton X-100, pH 7.6) for 45 min at room temperature. Sections were incubated overnight at 4°C with primary antibodies against Ki-67 or cleaved caspase-3 (both rabbit monoclonal anibody, Abcam; 1:500 dilution in blocking buffer). After washing in TBST, slides were incubated with HRP-conjugated goat anti-rabbit secondary antibody (Abcam; 1:1000) for 1 h. Immunoreactivity was visualized using 3,3′-diaminobenzidine, counterstained with hematoxylin, dehydrated, cleared in xylene, and mounted.

QUANTIFICATION AND STATISTICAL ANALYSIS

Statistical analysis

All data are presented as the mean ± standard error of the mean (SEM) unless otherwise stated. Statistical analyses were performed using GraphPad Prism (version 10.6.1, GraphPad Software, San Diego, CA, USA) with significance defined as $p < 0.05$ for all tests unless otherwise specified. For analysis with assay-specific statistical procedures, including bulk RNA-seq differential expression analysis, GO enrichment analysis, Bliss independence analysis, and IC_{50} curve fitting, statistical methods are described in the corresponding Methods sections and figure legends. Differences between two groups were evaluated using paired two-tailed Student's t tests. When comparing multiple groups, one-way analysis of variance (ANOVA) followed by Dunnett's post-hoc test was employed for comparisons against control group. If the data did not meet assumptions of normality or equal variance, the corresponding non-parametric tests (Mann-Whitney U test) were used. Exact values of n, definitions of n, statistical tests, and definitions of center and error bars are provided in the corresponding figure legends.

ADDITIONAL RESOURCES

Not applicable.

Cell Reports Medicine, Volume *7*

Supplemental information

Combine antibody-drug conjugate with cmLumiOpto

for triple-negative breast cancer treatment

Tanvi Varadkar, Zhuoxin "Zora" Zhou, Yingnan Si, Jiashuai Zhang, Zhantao Du, Anjana Gautam Khanal, Srijita Chowdhury, Chandini Tsaliki, Lufang Zhou, and Xiaoguang "Margaret" Liu

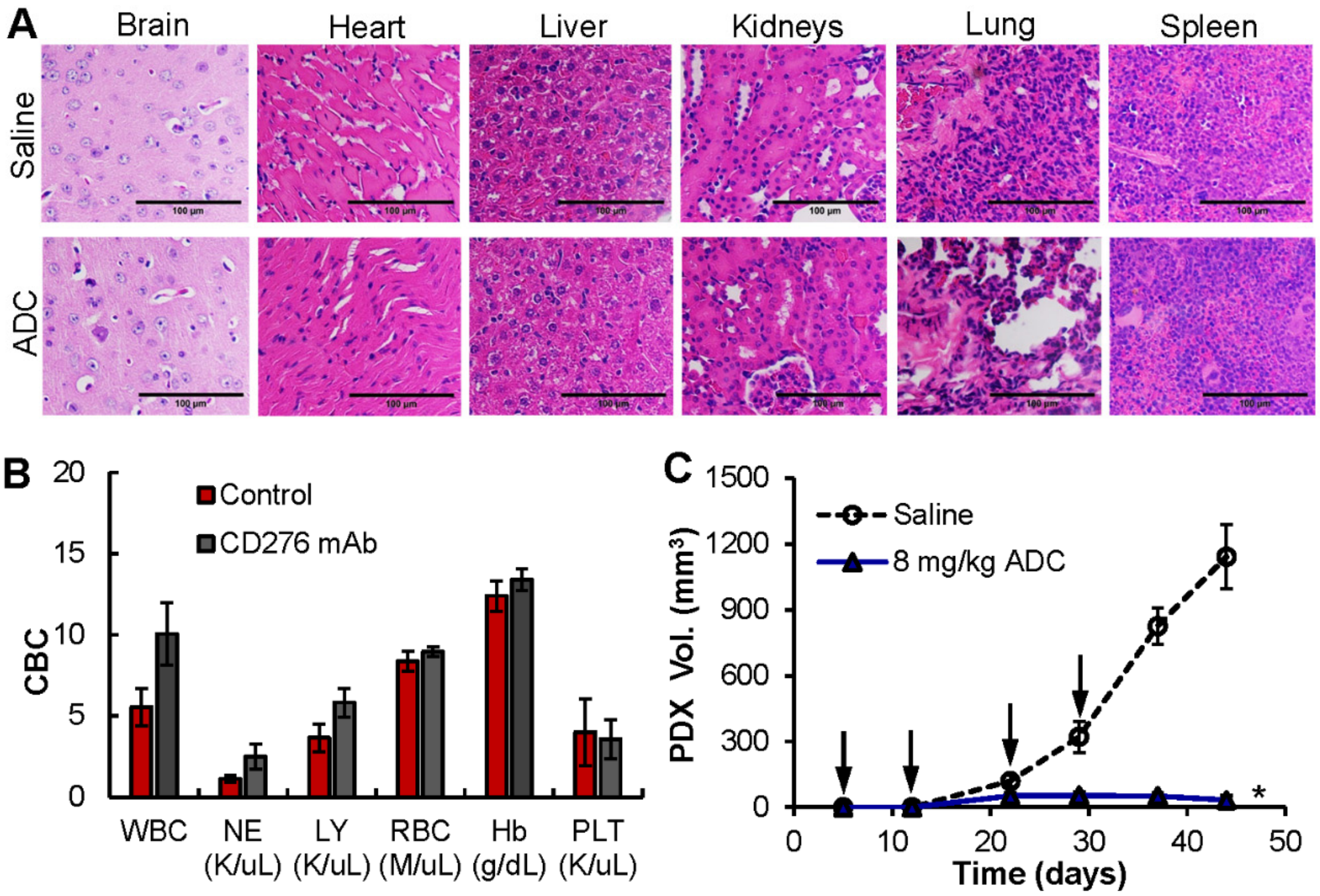

Figure S1. Evaluations CD276 mAb and ADC. Related to Figure 3. (**A**) H&E staining of major organs (brain, heart, liver, kidney, lung, and spleen) at 28 days post i.v. injection of ADC *via* tail vein in BALB/cJ mice. Scale bar: 100 µm. (**B**) Whole blood analysis showing minimal effect of CD276 mAb on blood cell count and peripheral immunity in BALB/cJ. n=4. Data are presented as mean±SEM. WBC: white blood cell; NE: neutrophils; LY: lymphocyte; RBC: red blood cell; Hb: hemoglobin; PTL: platelet. (**C**) PDX tumor volume profile. TNBC PDX xenografted female NSG mice treated with CD276 mAb-DM1 and saline following schedule of Q4Dx4 as indicated by the black arrow. n=7 mice/group. Data are presented as mean±SEM. *P<0.05 *vs*. saline using ANOVA followed by Dunnett's *t*-test.

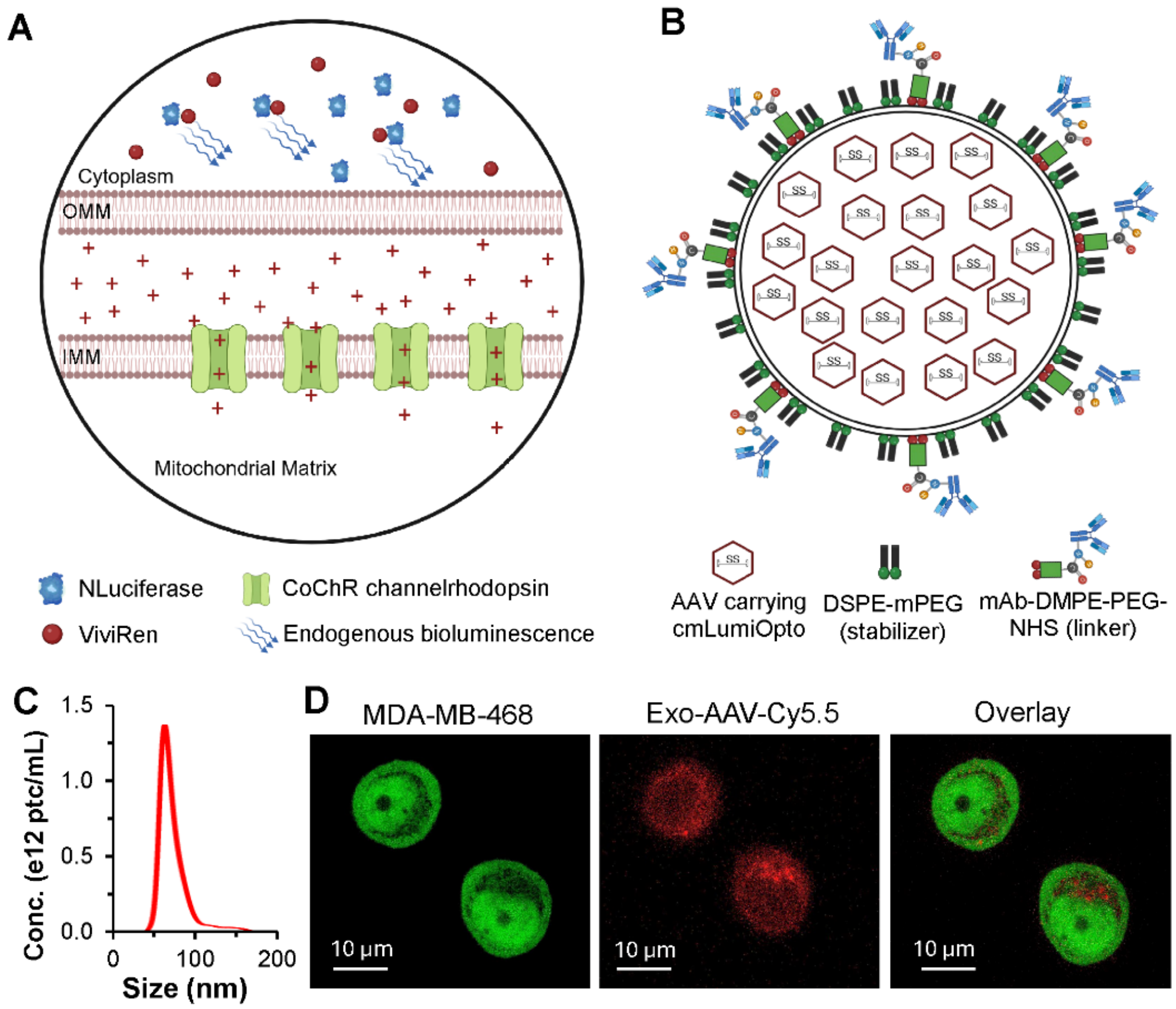

Figure S2. Construction and characterization of mAb-Exo-AAV. Related to Figure 4. (**A**) Diagram of the concept of cmLumiOpto gene therapy. cmLumiOpto gene vector leads to the expression of the CoChR protein with *cfos* promoter in the inner mitochondrial membrane of cancer mitochondria. ViviRen coupled with NLuc in cell cytoplasm generates a bright endogenous luminescent signal that activates the CoChR channels and causes depolarization of cancer mitochondria. (**B**) Structure of CD276 mAb-Exo-AAV which delivers cmLumiOpto gene. (**C**) Nanosight Pro analysis the concentration and particle size distribution of mAb-Exo-AAV. (**D**) Confocal image showing the internalization of mAb-Exo-AAV into MDA-MB-468 cells 24 hrs after incubation.

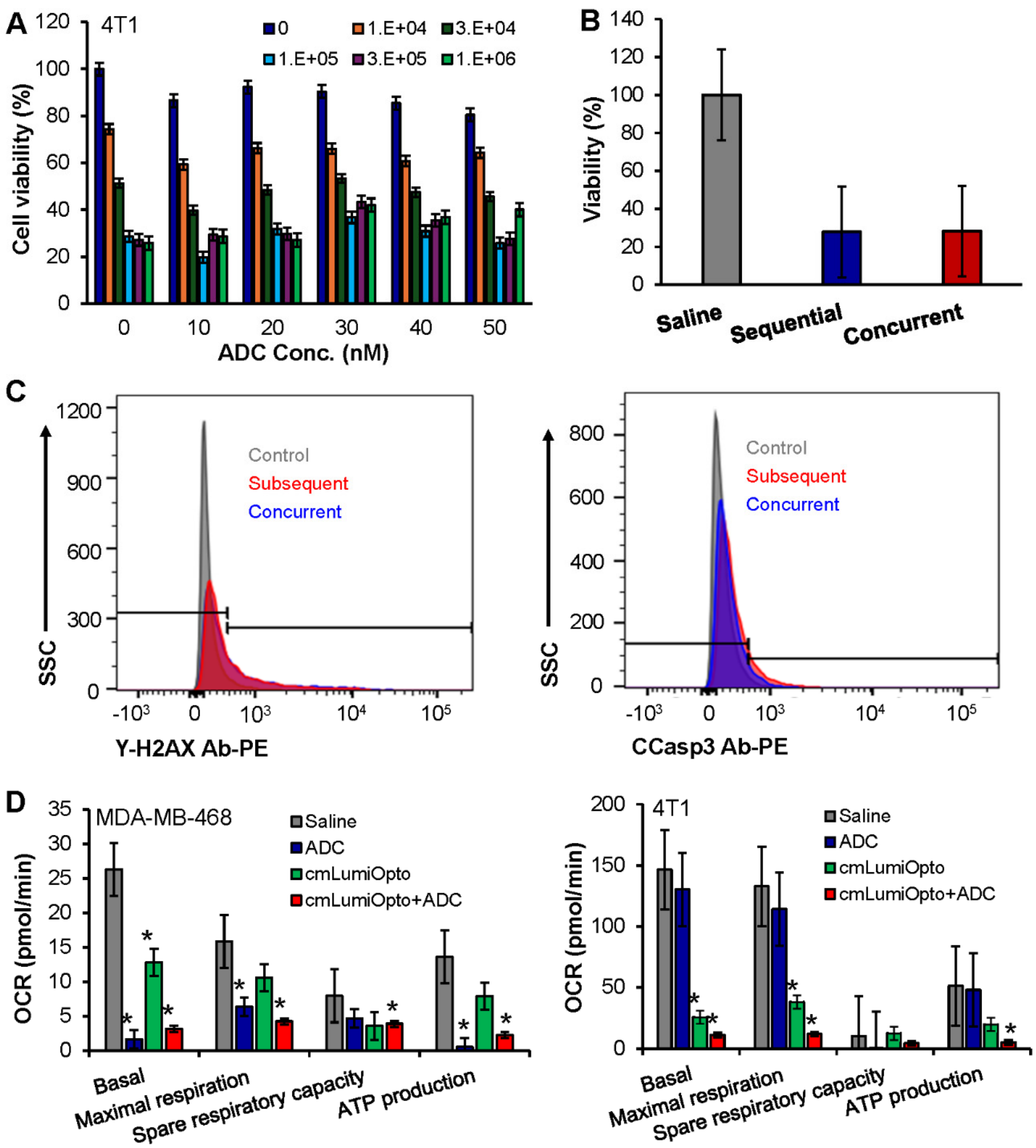

Figure S3. ***In vitro*** **assessment of synergism of cmLumiOpto and ADC. Related to Figure 4.** (**A**) Bliss independence assay in 4T1 cell line. n=3. Data are presented as mean±SEM. (**B**) Comparison of cell viability of sequential and concurrent treatment with 10 nM of ADC and $1x10^5$ MOI of cmLumiOpto in MDA-MB-231 cells. n=3. Data are presented as mean±SEM. (**C**) Analysis of the expression of apoptotic (cleaved caspase-3) or DNA damage (γ-H2AX) markers in sequential and concurrent treatments. (**D**) Seahorse assay to analyze mitochondrial function of MDA-MB-468 and 4T1 cells post treatment. n=4. Data are presented as mean±SEM.

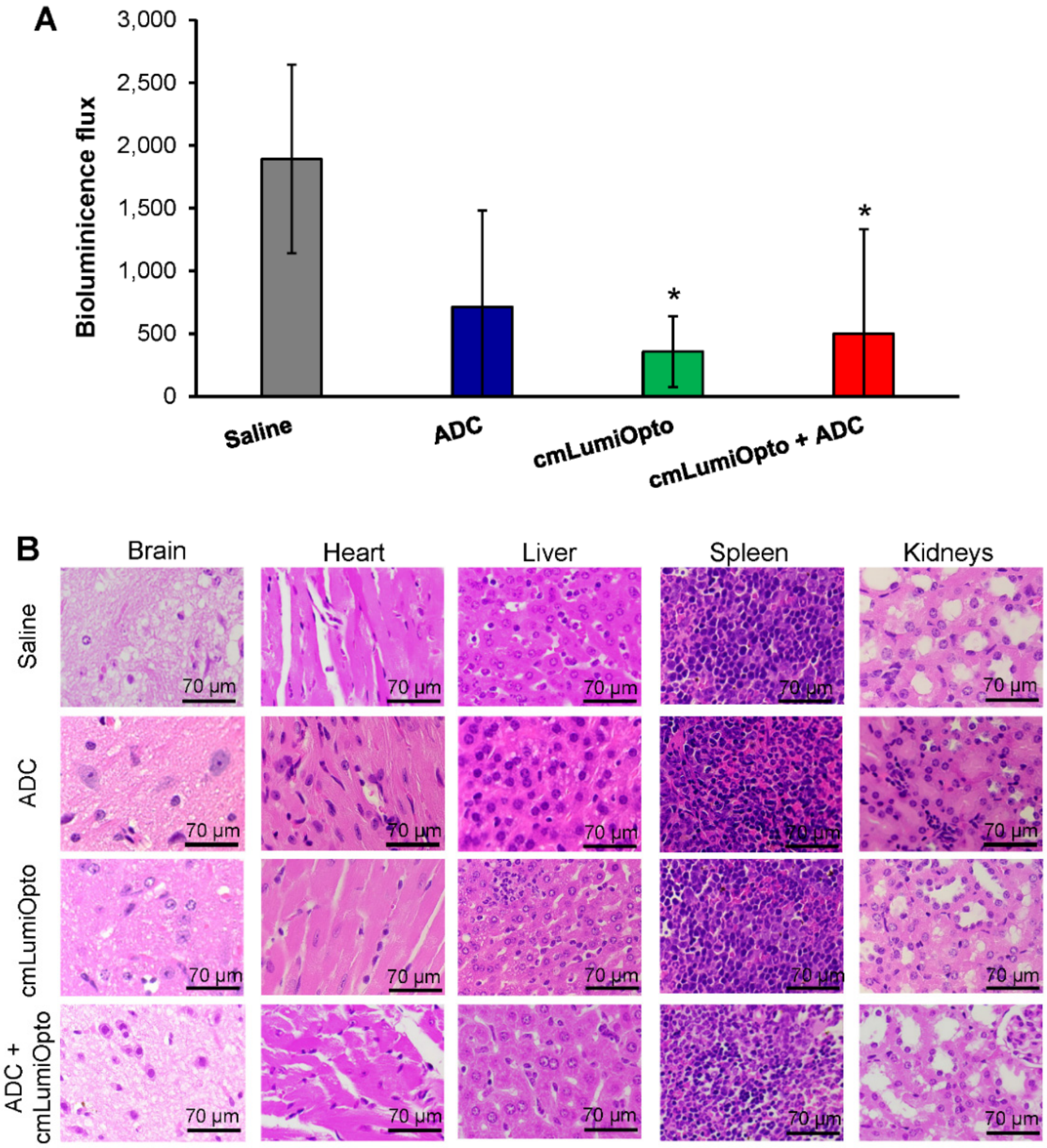

Figure S4. Potential toxicity evaluation in metastatic syngeneic models. Related to Figure 5. Mice were treated with saline (control), CD276 mAb-DM1 (8 mg/kg), cmLumiOpto ($10x10^{10}$ ptc/kg), and combination of ADC/cmLumiOpto (same dose as monotherapy). n=5 mice/group. Data are presented as mean±SEM. (**A**) Tumor bioluminescence flux of mice in saline treatment groups. **P*<0.005. (**B**) H&E staining of major organs harvested including brain, heart, liver, spleen and kidneys. Scale bar: 70 µm.

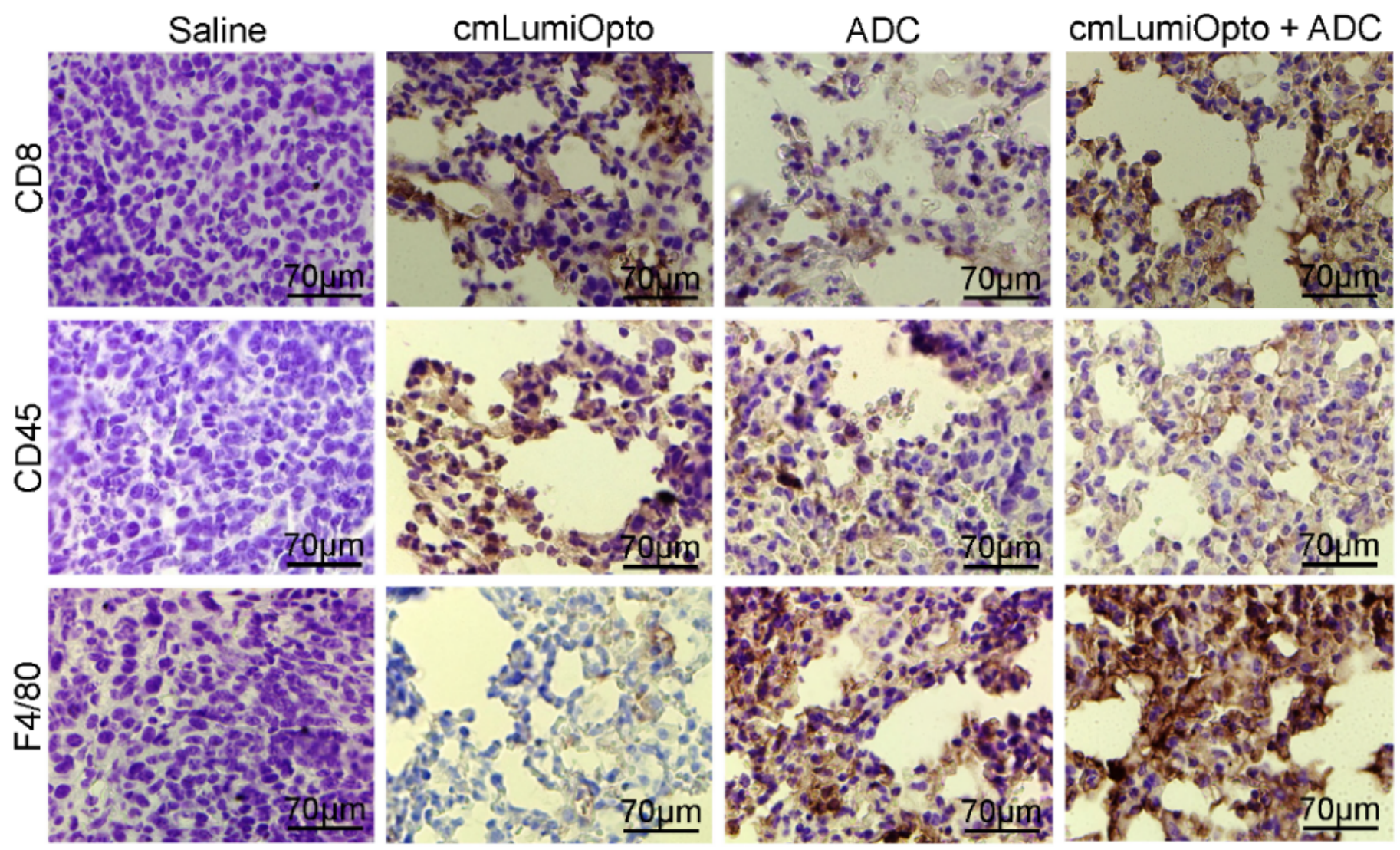

Figure S5. Immune activation evaluation in metastatic syngeneic models. Related to Figure 6. Mice were treated with saline (control), CD276 mAb-DM1 (8 mg/kg), cmLumiOpto ($10x10^{10}$ ptc/kg), and combination of ADC/cmLumiOpto (same dose as monotherapy). n=5 mice/group. IHC staining tumor slides with antibodies targeting CD8, CD45 and F4/80. Scale bar: 70 µm.
